# Hidden evolutionary constraints dictate the retention of coronavirus accessory genes

**DOI:** 10.1101/2023.10.12.561935

**Published:** 2023-10-14

**Authors:** Stephen A. Goldstein, Teagan M. Feeley, Kristina M. Babler, Zoë A. Hilbert, Diane M. Downhour, Niema Moshiri, Nels C. Elde

**Affiliations:** 1 Department of Human Genetics, University of Utah School of Medicine, Salt Lake City, UT, USA; 2 Howard Hughes Medical Institute, 4000 Jones Bridge Rd, Chevy Chase, MD 20815, USA; 3 Department of Computer Science and Engineering, University of California, San Diego, La Jolla, CA 92093

## Abstract

Genetic innovation is fundamental to the ability of viruses to adapt in the face of host immunity. Coronaviruses exhibit many mechanisms of innovation given flexibility in genomic composition relative to most RNA virus families^1–5^. Examples include the acquisition of unique accessory genes that can originate by capture of cellular genes or through duplication and divergence of existing viral genes^6,7^. Accessory genes may be influential in dictating viral host range and cellular tropism, but little is known about how selection acts on these variable regions of virus genomes. We used experimental evolution of mouse hepatitis virus (MHV) with an inactive native phosphodiesterase, NS2, that encodes a complementing cellular AKAP7 gene^9^, to simulate the capture of a host gene and found hidden patterns of constraint that determine the fate of coronavirus accessory genes. After courses of serial infection, AKAP7 was retained under strong selection but rapidly lost under relaxed selection. In contrast, the gene encoding inactive NS2, ORF2, remained intact, suggesting it is under cryptic evolutionary constraint. Guided by the retention of ORF2 and hints of similar patterns in related betacoronaviruses, we analyzed the evolution of SARS-CoV-2 ORF8, which arose via gene duplication^6^ and contains premature stop codons in several globally successful lineages. As with MHV ORF2, the coding-defective SARS-CoV-2 ORF8 gene remains largely intact, mirroring patterns observed during MHV experimental evolution and extending these findings to viruses currently adapting to humans. Retention of inactive genes challenges assumptions on the dynamics of gene loss in virus genomes and can help guide evolutionary analysis of emerging and pandemic coronaviruses.

## Introduction

Coronaviruses in the *Nidovirales* order have some of the largest known RNA genomes, ranging from ~27–32 kilobases^10,11^. The stability of these large genomes is facilitated by the innovation of a proofreading exonuclease into the RNA polymerase complex, unique among RNA viruses, that enhances replication fidelity^12–14^. Consequently, coronaviruses can accommodate addition of genetic material in the form of accessory genes acquired via horizontal gene transfer^8,15,16^ and gene duplication^6,17^. Evolutionary theory on the streamlining of RNA virus genomes^1,3,4^ predicts that accessory genes should encode near-immediate fitness benefits to avoid deletion.

Experimental studies of tobacco etch virus (TEV) support the idea that exogenous genetic material is rapidly purged under relaxed selection^2,3^. However, exogenous genes engineered into TEV, a small single-stranded positive sense RNA virus, substantially increase genome size, which might drive loss regardless of a potential fitness benefit offered by the gene product. To date, no comparable studies using animal RNA viruses to test the interplay between gain of beneficial coding sequence and selection on genome size are reported. Therefore, our understanding of how viral genome architecture and content evolve is poor, relative to the intense focus on how viruses evolve by nucleotide substitution. Given the major evolutionary leaps afforded by gene rearrangements, gains, and losses this represents a significant gap in our fundamental understanding of viral evolution.

To explore how selection acts on newly acquired viral genes we used experimental evolution of mouse hepatitis virus (MHV), a prototypical betacoronavirus, the genus that includes SARS and MERS-related coronaviruses. ORF2 of MHV and related viruses encodes a 2’−5’ phosphodiesterase (PDE), NS2, that antagonizes the OAS-RNase L antiviral pathway. We used recombinant MHV encoding a cellular PDE, the AKAP7 central domain (AKAP7), as a functional replacement for an inactive NS2^9^ with a substitution at position 126 (His126Arg), that results in restricted replication^18^. Viral PDEs were derived via capture of cellular AKAP7-like genes so using this virus, MHV^AKAP7^, simulates a horizontal transfer event giving rise to a novel viral gene. Critically, the AKAP7 PDE is inserted in place of ORF4^9^, allowing us to probe the relationship between selection and gene retention without introducing a substantial expansion of the genome.

We found that the retention of coronavirus accessory gene sequences in different selective environments does not conform to expectations of genome streamlining. Under relaxed selection in the absence of an active OAS-RNase L immune defense, AKAP7 was rapidly lost, consistent with selective pressure favoring reduced genome size. However, in striking contrast, MHV ORF2 was retained under all conditions tested. In parallel, SARS-CoV-2 ORF8 has been maintained largely intact even in lineages where it has lost coding capacity. These results suggest fundamental and widespread constraints on reducing viral genome size that may result from genes with overlapping coding and non-coding functions that influence coronavirus evolution.

## Results

### Cell type-dependent selective pressure on viral phosphodiesterases

**Figure 1.**
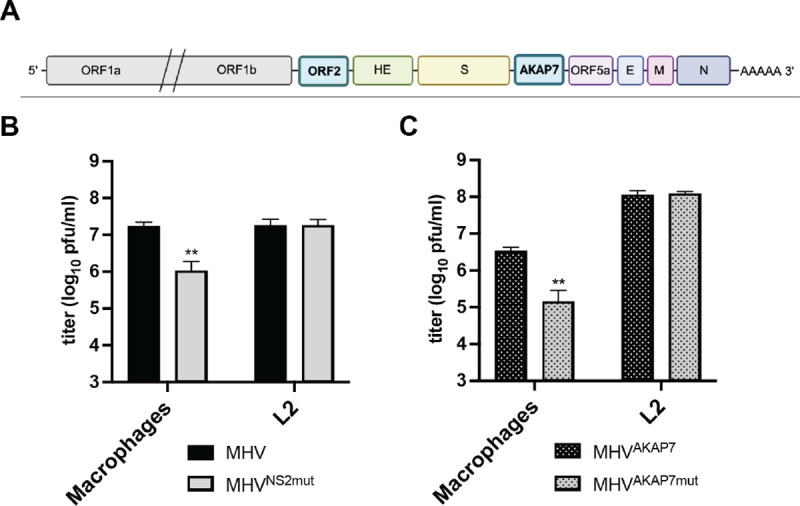
Cell-type specific selective pressure on MHV^AKAP7^. A) Schematic of the MHV genome with the AKAP7 PDE inserted in place of ORF4. B) Macrophages restrict replication of MHV^NS2mut^ 24 hours post-infection at MOI=0.01. p=0.0016. C) Macrophages but not L2s restrict replication MHV^AKAP7mut^, 24 hours post-infection at MOI=0.01. p=0.0018. Representatives of three independent experiments are shown. Statistical significance was determined by unpaired t-test.

To test outcomes of selection on coronavirus genome composition, we used a mouse hepatitis virus (MHV) variant that has an inactivating H126R amino acid change in NS2, its native PDE, which is functionally complemented by insertion of the coding sequence for the cellular AKAP7 PDE central domain (AKAP7)^9^ (Figure 1A). The AKAP7 gene inserted into MHV is 618 nucleotides and replaces the non-essential 321 nucleotide ORF4, so expansion of the genome is minimal (~1%). MHV with inactive NS2 (MHV^NS2Mut^) is restricted in mouse primary bone marrow-derived macrophages (BMDMs) by OAS-RNase L^18^, while AKAP7 inserted into MHV (MHV^AKAP7^) compensates for the inactive NS2 and restores replication to wild-type virus levels in BMDMs and mice^9^. MHV^NS2mut^ is restricted in immortalized primary macrophages^19^, but replicates to wild-type levels in L2 mouse fibroblasts (Figure 1B), providing differential selection for courses of experimental evolution^18^. Similarly, MHV^AKAP7^ replicated to high titers whereas MHV encoding an inactive AKAP7 PDE (MHV^AKAP7mut^) was significantly restricted (Figure 1C), demonstrating that these cells have an active OAS-RNase L pathway that imposes selective pressure to retain an active PDE. In contrast, MHV^AKAP7^ and MHV^AKAP7mut^ replicated to an equivalent level in L2 fibroblasts, establishing these cells as a model for MHV^AKAP7^ evolution under relaxed selection (Figure 1C).

### Deletion of AKAP7 occurs rapidly under relaxed selection

**Figure 2.**
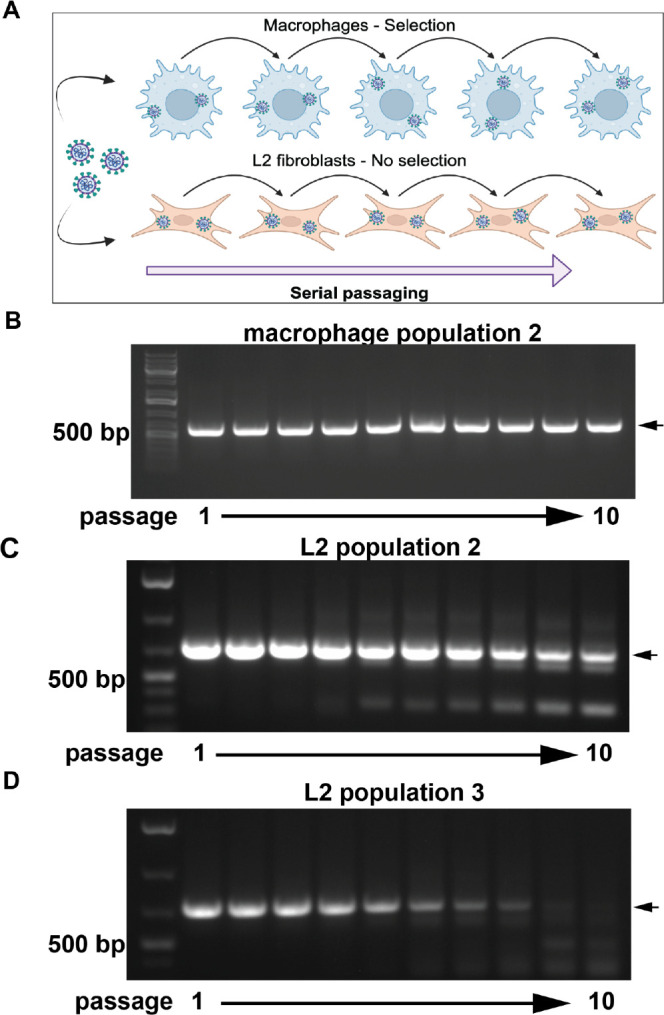
AKAP7 is rapidly lost during passage in L2 fibroblasts A) Schematic of the experimental evolution workflow. B) AKAP7 PCR showing retention of the full-length gene through ten passages in macrophages. C-D) PCR showing progressive deletion of AKAP7 during passage in L2 cells.

Our experimental evolution workflow uses serial passage in macrophages and L2 cells to model evolution of a horizontally acquired PDE under different conditions (Figure 2A). We plaque purified passage 0 (p0) MHV^AKAP7^ and conducted ten serial passages in triplicate in each cell type at a multiplicity of infection (MOI) of 0.01. At each passage we titrated virus production by plaque assay to calculate the MOI for the subsequent passage and collected total RNA for analysis of AKAP7 by PCR and Sanger sequencing. In macrophages, full-length AKAP7 was retained through ten passages (Figure 2B, Figure S1A–B), whereas in L2 cells, smaller PCR-generated bands appeared by passage two (Figure 2C, Figure S1C). The smaller bands became increasingly prominent through p10, while the full length AKAP7 band faded in intensity, indicating viruses with the full-length gene were disappearing from the population (Figure 2C–D). We cloned and sequenced full length bands from macrophages and L2 cells, and smaller bands from L2 cells at each passage. AKAP7 from both cell types contained no mutations (Supplementary File 1). The smaller bands replacing full-length AKAP7 during L2 passage revealed substantial deletions (Supplementary File 1). The AKAP7 deletion variants did not undergo nucleotide mutations, consistent with high fidelity coronavirus replication and revealed a lower barrier to genetic change by deletion than mutation, at least in the AKAP7 gene. Virus populations recovered at passage 10 from L2s exhibited significantly restricted replication relative to macrophage-passaged virus populations (Figure S2A), demonstrating that despite the heterogeneity in AKAP7 deletion patterns between replicates, all three MHV^AKAP7^ populations lost the ability to effectively suppress OAS-RNase L.

In addition, we plaque purified isolates from replicate three p10 virus populations from both cell types and used these isolates to infect macrophages and L2 cells. Consistent with the loss of AKAP7 during L2 passage, p10 plaque purified isolates from these cells exhibited significantly reduced replication in macrophages but replicated to wild-type titers in L2s, with some experiments showing a small growth advantage for AKAP7-deleted isolates, which warrants further study (Figure S2E–G). The rapid loss of AKAP7 under relaxed selection is consistent with an evolutionary model wherein new viral genes must provide a sufficient near-immediate advantage to be retained long enough for further adaptation.

### Inactive MHV ORF2 is retained under strong and relaxed selection

**Figure 3.**
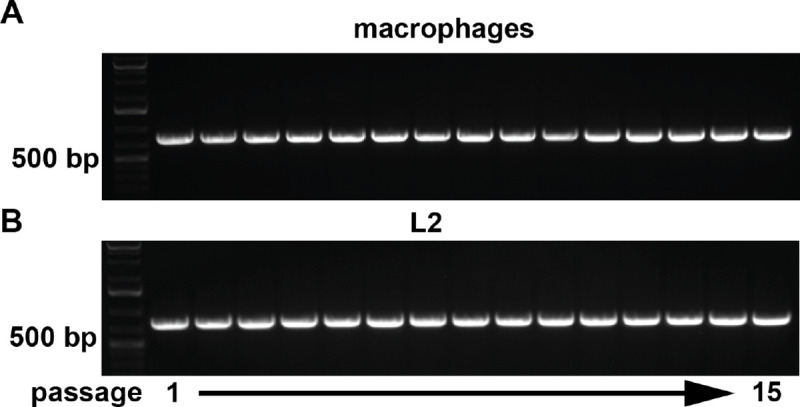
Retention of ORF2 during serial passage in both cell types. A) ORF2 PCR of passage 1–15 virus populations in macrophages. B) ORF2 PCR of passage 1–15 virus populations in L2 cells. C-F) Direct cDNA relative coverage plots of ORF2 in plaque purified virus isolates from p0, macrophage p10, and representative L2 p10 showing retention of ORF2.

Like the loss of AKAP7 under relaxed selection, there is a strong *a priori* prediction that ORF2, which encodes an inactive PDE in MHV^AKAP7^, would be also rapidly lost from the MHV genome. Unlike AKAP7, ORF2 with the H126R inactivating substitution should be dispensable in both macrophages and L2 fibroblasts. Surprisingly, PCR of ORF2 (Figure 3A–B) at each serial passage in macrophages and L2s and sequencing of ORF2 after 10 passages, revealed no deletions, gene loss, fixed mutations, or reversions of the H126R substitution (Supplementary File 2). Given the unexpected retention of inactive ORF2, we conducted five additional passages in both cell types to provide additional opportunity for deletion. We sequenced the macrophage-passaged viruses again at p15 to confirm there was no amino acid 126 reversion that might suggest ORF2 retention in these cells was due to OAS-RNase L-mediated selection (Figure 3A–B; Supplementary File 2). The striking retention of ORF2 raised the possibility that protein-coding coronavirus genes can be under hidden evolutionary constraint in addition to selection on protein function.

To investigate if similar patterns of constraint exist in the wild, we analyzed ORF2 from other betacoronaviruses in the same subgenus as MHV. Rabbit coronavirus HKU14^20^ has a premature stop codon mutation truncating NS2 to 43 amino acids. However, the entire nucleotide sequence encoding full-length NS2 is present in 4/5 HKU14 sequences in NCBI, while the other has ~100 bp of missing sequence relative to human coronavirus OC43, one of its closest relatives (Supplementary FIle 3). In contrast, ORF2 in porcine hemagglutinating encephalomyelitis virus (PHEV) appears much more evolutionarily malleable^21–24^, with various isolates containing ORF2 with premature stop codons or deletions (Supplementary File 3). These findings are broadly consistent with constraints on deletion of ORF2, with the heterogeneity in PHEV suggesting there are ecological contexts in which even these widespread hidden constraints are relaxed.

### Long read sequencing confirms retention of ORF2 after experimental evolution

**Figure 4.**
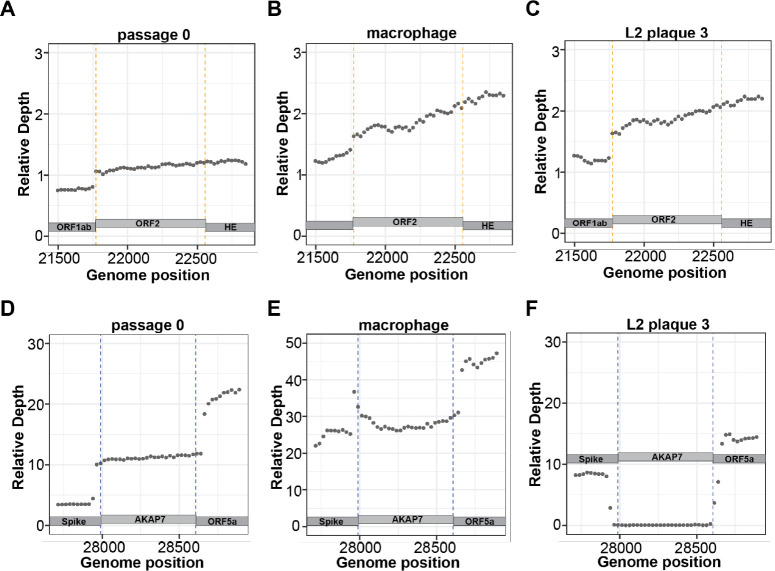
Direct cDNA sequencing analysis of AKAP7 and ORF2 evolution A-C) Relative coverage depth plots of ORF2 in purified plaque isolates from passage 0 MHV^AKAP7^, and passage 10 L2 fibroblast and macrophages purified plaques. B-E) Relative coverage depth plots of AKAP7 in purified plaque isolates from passage 0 MHV^AKAP7^, and passage 10 L2 fibroblast and macrophages purified plaques.

To fully characterize changes in the ORF2 and AKAP7 genes after experimental evolution we performed Oxford Nanopore direct cDNA sequencing of plaque purified MHV^AKAP7^ p0, five L2 p10 isolates and one macrophage p10 isolate. Mean read length across sequencing runs ranged from 1,065 to 1,528 bases and average coverage was 2,603x to 27,296x per nucleotide of the MHV^AKAP7^ genome with a mean of 8,943x. Across all sequencing runs ~70–75% of reads aligned to the MHV^AKAP7^ genome (Supplementary File 5). To quantify changes in ORF2 and AKAP7 gene content after ten passages and control for variable overall sequencing depth across runs, we normalized average coverage of ORF2 and AKAP7 to average coverage of ORF1ab for each isolate. Consistent with PCR analysis, average relative coverage of ORF2 was the same in plaque purified passage 10 isolates from macrophages or L2 fibroblasts (Figure 4A–C; Figure S3C–F), demonstrating with exquisite granularity that this genomic region is under previously hidden evolutionary constraint. The average relative AKAP7 coverage for p0 and macrophage p10 were 11.2 and 27.8, respectively, possibly reflecting enhanced synthesis of subgenomic mRNA following passage in macrophages, which could increase production of immune-modulating proteins and select for retention of AKAP7 during passage under OAS-RNase L selection. (Figure 4D–E). In contrast, the average relative coverage for L2 p10 isolates was 0.332, a 33-fold decrease from p0 consistent with PCR analysis. (Figure 4A–B, 4F; S3G–J).

### SARS-CoV-2 ORF8 is retained despite loss of coding capacity

Our analysis of ORF2 in MHV-like viruses suggested the constraints we identified experimentally also exist in wild circulating viruses. However, the power of this analysis was limited by the relative paucity of genomic data available for animal viruses. In contrast, the SARS-CoV-2 dataset curated in the Global Initiative on Sharing All Influenza Data (GISAID) database contains more than 16 million genomes, offering an unprecedented window into viral evolution. Analogous to the inactivation of MHV ORF2 in our system, SARS-CoV-2 ORF8 has acquired premature stop codons in numerous globally successful lineages, including B.1.1.7, XBB.1, XBB.1.5, XBB.1.9, and XBB.1.16., as anecdotally described. ORF8 has been of interest since early in the SARS-CoV-2 pandemic due to a small deletion that emerged in its SARS-CoV ortholog in 2003^25^, an early cluster of infections in Singapore involving an ORF8 deletion variant^26,27^, as well as debate over its role during SARS-CoV-2 infection^6,27–34^. Despite the intense focus on ORF8 function and loss of full-length protein synthesis, very little attention has been devoted to the evolution of the gene itself. Given the recurrent acquisition of premature stop codons and the position of ORF8 upstream of nucleocapsid, a key structural gene, we analyzed patterns of sequence retention.

**Figure 5.**
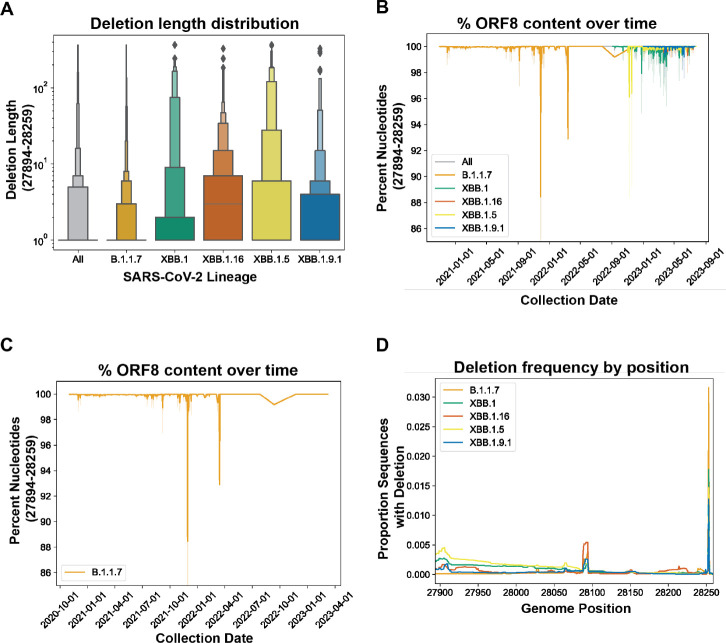
ORF8 retention in SARS-CoV-2 lineages B.1.1.7, XBB.1, XBB.1.5, XBB.1.9, and XBB.1.16 that have acquired early stop codon mutations. A) ORF8 deletion length distribution. B) ORF8 percent nucleotide content in SARS-CoV-2 plotted over time. C) ORF8 percent nucleotide content in SARS-CoV-2 lineage B.1.1.7 D) Proportion of SARS-CoV-2 sequences in indicated lineages with deletions at each position within ORF8.

Although deletion of ORF8 might be predicted based on loss of its protein function, we hypothesized that, given its positioning upstream of a key structural gene, that it would be retained. We downloaded all sequences assigned to the B.1.1.7, XBB.1, XBB.1.5, XBB.1.9, and XBB.1.16 lineages from GISAID and applied a cutoff date to exclude any samples with associated metadata suggesting they are incorrectly assigned. (Figure S4). We calculated a deletion length distribution of ORF8 in B.1.1.7 (45,920 sequences), XBB.1 (627 sequences), XBB.1.16 (518 sequences), XBB.1.5 (4,373 sequences), and XBB.1.9.1 (425 sequences) (Figure 5A), finding that across all sampled lineages 96% of deletions were smaller than ten nucleotides, ranging from a high of 97.6% for B.1.1.7 to a low of 81.8% for XBB.1.5. (Supplementary File 5). We then calculated the percent of ORF8 gene content in these lineages, sorted by collection date, and plotted these values for all genomes from all five lineages together (Figure 5B) and individually (Figure 5C; Figure S5). Genomes with less than 95% ORF8 gene content were rare, and in XBB.1-derived lineages with a greater percent of genomes with large deletions these genomes were collected primarily early or in the middle of the circulation period of these variants rather than increasing at later collection dates as would be expected if ORF8 was being deleted over time (Figure 5C; Figure S5). Finally, we calculated deletion frequency by position for all five lineages and observed two peaks, one in the middle of the gene and one at the 3’ end, but deletions were otherwise uniformly rare (Figure 5D) These results are consistent with the retention of the ORF8 gene promoting SARS-CoV-2 fitness independent of selection on protein function.

## Discussion

Due to their large genome sizes compared to other RNA viruses, coronaviruses are amenable to horizontal gene transfer and gene duplications, among various mechanisms of genomic innovation and adaptation. They also exhibit signatures of constraint or streamlining of genome size, such as a ribosomal frameshift in ORF1ab as well as overlapping reading frames and bicistronic subgenomic RNAs observed among diverse coronaviruses^35^. How selection acts to balance adaptations involving genomic expansions and reductions, particularly in RNA viruses, is largely uncharacterized.

Findings from this study challenge a simple evolutionary model wherein the fate of protein coding genes hinges entirely on protein function. The persistent, unexpected retention of ORF2 in both macrophages and L2 fibroblasts through fifteen serial infections suggests a complex interplay of selective pressure acting on viral coding sequence. In L2s the PDE activity of ORF2 is dispensable, and the stability of the H126R substitution during experimental evolution in macrophages is consistent with AKAP7 fully counteracting OAS-RNase L activity. While it is formally possible that an unknown secondary role for the ORF2-encoded protein NS2 antagonizes innate immunity, no such function has been identified and the protein contains no identifiable domains other than the PDE.

The difference in genomic location between ORF2 and AKAP7 may be relevant to their respective fates. AKAP7 is located upstream of ORF5a, which encodes an accessory gene with unknown function, whereas ORF2 immediately precedes the hemagglutinin-esterase (HE) and spike structural genes. HE subgenomic mRNA is not expressed from the MHV A59 genome^36,37^, suggesting that some element within the ORF2 sequence or merely its presence may impact spike subgenomic mRNA synthesis and enhance viral fitness. ORF2 was lost in the HCoV-HKU1 (Supplementary File 3) lineage, but other viruses descended from the HKU1 zoonotic reservoir have not been sampled, precluding a historical analysis of this deletion event. Determining whether ORF2 loss occurred before or after spillover into humans could provide important clues about selection acting on genome composition soon after zoonosis.

Direct parallels between the experimental retention of ORF2 and the recurrent retention of coding-defective SARS-CoV-2 ORF8 highlights the power of laboratory evolutionary studies for detecting patterns of constraint on pandemic viruses. Under a conventional model of selection favoring genome streamlining, viruses with ORF8 deleted would rapidly dominate B.1.1.7 and XBB.1-derived lineages, undergoing selective sweeps soon after the potential to encode full-length protein was lost. Analogous to the genomic position of ORF2 upstream of a key structural gene, ORF8 is directly 5’ of nucleocapsid. Previous work showed that changes in nucleocapsid can enhance viral fitness^38^, and the retention of ORF8 indicates that it plays a role in SARS-CoV-2 fitness irrespective of the function of the protein it encodes, which seems to explain widespread patterns of ORF8 sequence retention.

The exact nature of evolutionary constraint on MHV ORF2 and SARS-CoV-2 ORF8 is not yet clear. An intriguing possibility is that the retained RNA sequence encodes a regulatory function tuning transcription of other genes. MHV ORF2 is upstream of spike, albeit with the defective HE gene intervening and SARS-CoV-2 ORF8 immediately precedes nucleocapsid. If accessory genes promote synthesis of spike and nucleocapsid subgenomic mRNAs, viral fitness could be negatively impacted by reduced transcription and the resulting decrease in protein synthesis. Such regulatory functions might be sequence specific, involve RNA-RNA interactions, or relate to RNA secondary structure. While one study suggested RNA-RNA interactions between SARS-CoV-2 ORF8 and spike genes^39^, these possibilities are not well-characterized.

In future work, identifying patterns of evolutionary constraint may serve as an indicator of hidden sequence features impacting viral fitness. These regions can be prioritized for mechanistic studies using reverse genetics systems of MHV and SARS-CoV-2, opening a new bridge between experimentation and computational analysis of viral evolution. Experimentally dissecting fitness impacts will likely require the use of competition studies that can reveal subtle adaptive shifts in replication. The loss of ORF2 in HCoV-HKU1 demonstrates that viruses lacking this gene are viable, so any fitness defects of MHV^ΔORF2^ might appear only over courses of experimental evolution or in direct competition with wild-type viruses. By identifying features under selection with experimental evolution to guide searches in genomic databases of viral populations, a new door opens to discovery of previously hidden viral evolutionary dynamics and constraints.

## Materials and Methods

### Cells and Viruses

Immortalized primary macrophages were provided by Sunny Shin^19^, 17-Clone 1 and L2 cells were provided by Susan Weiss. Macrophages were cultured in RPMI-1640 supplemented with 10% FBS, 1% L-glutamine, and 1% Penicillin/Streptomycin, while 17-Clone 1 and L2 cells were cultured in DMEM supplemented the same way.

All viruses used in this study were provided by Susan Weiss at the University of Pennsylvania. Wild-type MHV and MHV^NS2mut^ were previously described^18^ as were MHV^AKAP7^ and MHV^AKAP7mut9^. Viruses were received as seed aliquots and 100 μl of each virus was added to one T75 flask containing a confluent monolayer of 17Cl1 cells in 2 ml volume. Flasks were incubated for one hour at 37° C, after which 10 ml of fresh media was added. Once cytopathic effect was present (18–24 hours post-infection) the flasks were put through three freeze-thaw cycles and the supernatant was clarified via centrifugation and aliquoted for later use.

### Virus infections and plaque assays

One day after seeding in 12-well plates macrophages or L2 cells were infected at an MOI of 0.01 in 200 μl total volume and incubated for one hour at 37 ° C with rocking every 15 minutes. After one hour cells were washed 3 times with PBS and 1 ml of fresh media (2% FBS) was added to each well. 24 hours post-infection 300 μl of supernatant was collected and stored at −80° C. We also harvested total RNA for additional analyses (described below). Initial infections, all serial passages, and endpoint replication comparisons were done at an MOI of 0.01. Viral titers were calculated by plaque assay on confluent L2 cell monolayers. Supernatants collected 24 hours post-infection were serially diluted 1:10 to 10^−8^, inoculated onto L2 monolayers and incubated for 1 hour at 37 ° C with rocking every 15 minutes. After one hour 3 mL of semi-solid agarose overlay was added to each well and the plates returned to 37 ° C for 24 hours. 24 hours post-infection the agarose overlay was removed and the monolayers were washed with PBS and stained with 0.5% crystal violet plus 20% methanol. The stain was washed off with water and plaques counted and recorded.

### RNA extraction and cDNA synthesis

Total RNA was harvested 24 hours post-infection on each serial passage with the Zymo Research Quick-RNA Miniprep Kit (Cat. #R1057) following the manufacturer’s protocol. To increase yield we eluted the RNA in a reduced volume of 25 μl nuclease-free water. RNA quantification and quality assessment was conducted using a Synergy HT BioTek plate reader. cDNA was synthesized using the Thermo Scientific Maxima First Strand cDNA Synthesis Kit with dsDNase (Lot. 2664799) with 1 μg of input RNA.

### PCR and Sanger sequencing

On each serial passage the AKAP7-CD and ORF2 genes from MHV^AKAP7-CD^ were analyzed by PCR. We used Phusion Flash HiFi Master Mix (Cat # F548S ) in 20 μl/reaction with 2 μl of cDNA template. Thirty cycles of PCR were performed. Cycling conditions were: **1)** Denaturation, 98°C for 10 seconds **2) a)** Denaturation (98°C for 2 seconds) **b)** Annealing (63°C for 10 seconds) **c)** Extension (72°C for 30 seconds) **d)** Final extension (72°C for 2 minutes) **3)** Hold (4°C).

PCR products were visualized by agarose gel (1%) electrophoresis then extracted and purified using Zymoclean Gel DNA Recovery Kit (Zymo Research Lot. #213587) following manufacturer protocols. DNA was eluted in 10 μl. Purified PCR products were prepared for Sanger Sequencing by TOPO cloning using the following protocol: **1) Addition of polyA-tail:** Incubate at 72°C for minutes in 12 μl total volume including 10x Standard Taq Reaction Buffer (NEB Cat. #B9014S), dATP (NEB Cat. #N0440S), Taq DNA Polymerase (NEB Cat. #M0273L), and nuclease-free water. **2) Insertion into TOPO vector:** Incubate 4 μl polyA-tailed PCR product at room temperature for 30 minutes with 1 μl pCR 2.1-TOPO vector (Invitrogen Lot. # 2392593) and 1 μl salt solution (Invitrogen Lot. #2376907). **3) Transformation into DH5ɑ *E. coli:* a)** Add 6 μl TOPO reaction to 100 μl DH5ɑ **b)** Incubate on ice for 30 minutes **c)** Heat shock at 42°C for 30 seconds **d)** Place on ice for 2 minutes **e)** Add 250 μl SOC media **f)** Shake at 225 rpm at 37°C for 2 hours **g)** Plate on LB + Carb agar plates pre-treated with Xgal (20 mg/mL) and IPTG (100 mM) for blue-white selection and incubate overnight at 37°C. **h)** Grow white colonies overnight in 5 ml LB+Carb with shaking at 225 rpm at 37°C **i)** Extract plasmid DNA using the Zippy Plasmid Miniprep Kit (Zymo Research Cat. # 11–308) following manufacturer protocol with elution 75 μl nuclease-free water. Plasmid DNA was quantified with a Synergy HT BioTek plate reader and sent to the University of Utah DNA sequencing core or Genewiz.

### Sequencing primers

**Table:** 

Primers	Forward	Reverse
AKAP7	5’-**ATTGCTACCTGGCCCCG**-3’	5’-**CTAGGGTCTTAGGCCCAAATG**-3’
MHV ORF2	5’-**ATGGCCTTTGCTGACAAGCCTAAT**-3’	5’-**TCAACACATACAACCCTTCATTCT**-3’
M13	5’-**GTAAAACGACGGCCAG**-3’	5’-**CAGGAAACAGCTATGAC**-3’

### Sequence analysis

Sequence files were imported into Geneious and aligned to either the AKAP7 or ORF2 coding sequences using the MAFFT Geneious plug-in ^40^. HKU1, HKU14, and PHEV multiple sequence alignments were likewise generated using the MAFFT Geneious plug-in with default parameters.

### Oxford Nanopore direct cDNA sequencing

1 mL of purified plaque was used to infect 2× 10 cm dishes of desired cell type (macrophages or L2). 18–20 hours post-infection total RNA was collected using Zymo Research Quick-RNA Miniprep Kit (Cat. #R1057). The same lysis buffer (1 ml) was used on both dishes/per infection to maximize yield. Polyadenylated RNA was extracted using the Invitrogen Poly(A)Purist MAG Kit (Cat # AM1922) following the manufacturer protocol. 100 ng PolyA+ RNA was prepared for sequencing using the Direct cDNA Sequencing Kit SQK-DCS109 from Oxford Nanopore Technologies, according to the manufacturer’s instructions. The prepared cDNA libraries were sequenced using the MinION Mk1C with FLO-MIN106D flow cells, version 9.4.1. Fastq files were generated from fast5 files using the high-accuracy model of Guppy basecaller, version 6.5.7 with the following parameters hac_guppy -c dna_r9.4.1_450bps_hac.cfg -x auto --compress_fastq.

### Direct cDNA sequencing coverage analysis

Fastq files from Nanopore sequencing runs were individually aligned to the MHV^AKAP7^ (passage 0) reference genome. Alignments were performed using minimap2 (v.2.23) and subsequently sorted and indexed with samtools (v.1.16). Coverage at every position across the genome for each sample was calculated using the bedtools (v.2.26.0) genomecov command. Coverage was then normalized for each sample to the average coverage across ORF1ab (nucleotide positions 211–21746 in the reference genome), which contains the non-amplified reading frame, allowing for comparison across sequencing runs and samples. A rolling average of this normalized read depth was calculated across the genome in 25 bp windows and plotted with ggplot2 in R. For AKAP7 and ORF2 analysis, rolling averages were recalculated for the region containing the gene sequence ± 300 bp of flanking sequence on either side and plotted identically to the genome wide coverage plots.

### SARS-CoV-2 ORF8 Deletion Analysis

All methods and code used to conduct this analysis are available at https://github.com/niemasd/SC2-Deletion-Analysis.

## Supplementary Material

Supplement 1

Supplement 2
