# Hidden evolutionary constraints dictate the retention of coronavirus accessory genes

**DOI:** 10.1101/2023.10.12.561935

**Published:** 2024-03-19

**Authors:** Stephen A. Goldstein, Teagan M. Feeley, Kristina M. Babler, Zoë A. Hilbert, Diane M. Downhour, Niema Moshiri, Nels C. Elde

**Affiliations:** 1 Department of Human Genetics, University of Utah School of Medicine, Salt Lake City, UT, USA; 2 Howard Hughes Medical Institute, 4000 Jones Bridge Road, Chevy Chase, MD 20815, USA; 3 Department of Computer Science and Engineering, University of California, San Diego, La Jolla, CA 92093,

## Abstract

Coronaviruses exhibit many mechanisms of genetic innovation^1–5^, including the acquisition of accessory genes that originate by capture of cellular genes or through duplication of existing viral genes^6,7^. Accessory genes influence viral host range and cellular tropism, but little is known about how selection acts on these variable regions of virus genomes. We used experimental evolution of mouse hepatitis virus (MHV) encoding a cellular AKAP7 phosphodiesterase and an inactive native phosphodiesterase, NS2 (ref 8) to simulate the capture of a host gene and analyze its evolution. After courses of serial infection, the gene encoding inactive NS2, ORF2, unexpectedly remained intact, suggesting it is under cryptic constraint uncoupled from the function of NS2. In contrast, AKAP7 was retained under strong selection but rapidly lost under relaxed selection. Guided by the retention of ORF2 and similar patterns in related betacoronaviruses, we analyzed ORF8 of SARS-CoV-2, which arose via gene duplication^6^ and contains premature stop codons in several globally successful lineages. As with MHV ORF2, the coding-defective SARS-CoV-2 ORF8 gene remains largely intact, mirroring patterns observed during MHV experimental evolution, challenging assumptions on the dynamics of gene loss in virus genomes and extending these findings to viruses currently adapting to humans.

## Introduction

Coronaviruses have unusually large RNA genomes, ranging from ~27–32 kilobases^9,10^. The stability of these large genomes is facilitated by the incorporation of a proofreading exonuclease into the RNA polymerase complex, unique among RNA viruses, that enhances replication fidelity^11–13^. Consequently, coronaviruses can accommodate addition of genetic material in the form of accessory genes acquired via horizontal gene transfer^14–16^ and gene duplication^6,17^. Evolutionary theory on the streamlining of RNA virus genomes^1,3,4^ predicts that new genes, such as coronavirus accessory genes, should encode near-immediate fitness benefits to avoid deletion.

Experimental studies of tobacco etch virus (TEV) support the assumption that exogenous genetic material is rapidly purged under relaxed selection^2,3^. However, exogenous genes engineered into TEV, a small single-stranded positive sense RNA virus, substantially increase genome size, which might drive loss regardless of a potential fitness benefit offered by the gene product. To date, no comparable studies using animal RNA viruses to test the interplay between gain of beneficial coding sequence and selection on genome size have been reported. Therefore, our understanding of how viral genome architecture and content evolve is poor, relative to the intense focus on how viruses evolve by nucleotide substitution. Given the major evolutionary leaps afforded by gene rearrangements, gains, and losses this represents a significant gap in our fundamental understanding of viral evolution.

To explore how selection acts on newly acquired viral genes we used experimental evolution of mouse hepatitis virus (MHV), a prototypical betacoronavirus, the genus that includes SARS and MERS-related coronaviruses. ORF2 of MHV and related viruses encodes a 2’-5’ phosphodiesterase (PDE), NS2, that antagonizes the OAS-RNase L antiviral pathway. We used recombinant MHV encoding a cellular PDE, the AKAP7 central domain (AKAP7), as a functional replacement for an inactive NS2 (ref 8) with a substitution at position 126 (His126Arg), that results in restricted viral replication^18^. Viral PDEs were derived via capture of cellular AKAP7-like genes^19,20^ so using this virus, MHV^AKAP7^, simulates a horizontal transfer event giving rise to a novel viral gene. Critically, the AKAP7 PDE is inserted in place of ORF4 (ref 8), allowing us to probe the relationship between selection and gene retention without substantial expansion of the genome.

We found that the retention of coronavirus accessory gene sequences in different selective environments does not conform to expectations of genome streamlining. Under relaxed selection in the absence of active OAS-RNase L immune pressure, AKAP7 was rapidly lost, consistent with the impact of selective constraints on genome size. However, in striking contrast, MHV ORF2 was retained under all conditions tested, despite encoding an inactive PDE. In parallel, we found that ORF8 of SARS-CoV-2 is largely intact even in lineages where it has lost coding capacity^21^. These results suggest fundamental and widespread constraints on streamlining of viral genomes due to genetic information with hidden functions beyond protein coding capacity.

## Results

### Cell type-dependent selective pressure on viral phosphodiesterases

**Figure 1.**
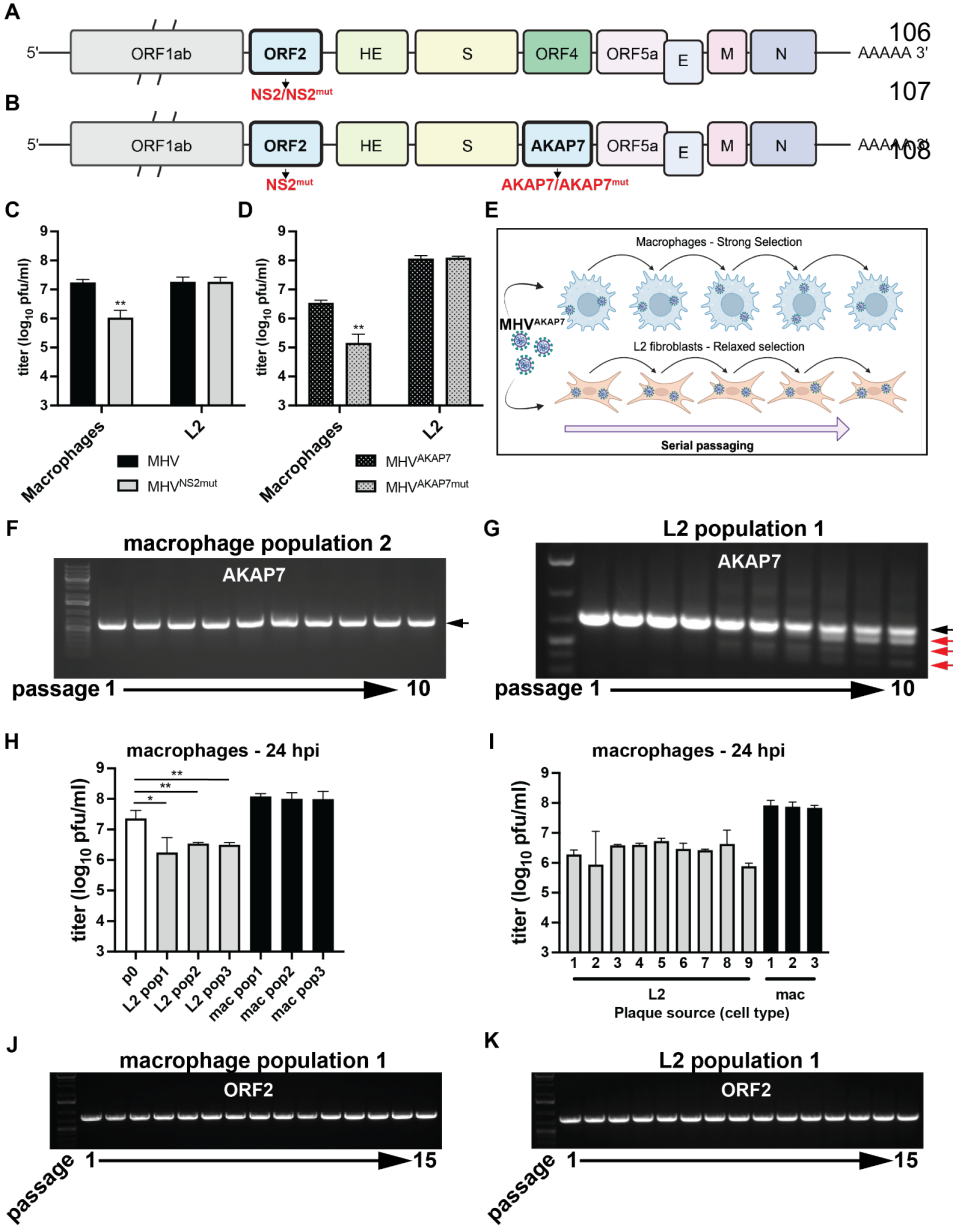
Experimental evolution of mouse hepatitis virus reveals hidden selective constraints. A) Schematic of the MHV genome with NS2, the protein encoded by ORF8 indicated. B) Schematic of the MHV^AKAP7^ genome, with AKAP7 inserted in place of ORF4. C) Replication of wild-type MHV and MHV^NS2mut^ in macrophages (p=0.002) and L2 fibroblasts (p=0.968) 24 hpi, infected at an MOI of 0.01. One representative experiment of three is shown. D) Replication of MHV^AKP7^ and MHV^AKAP7mut^ in macrophages (p=0.002) and L2 cells (p=0.681) 24 hpi, infected at an MOI of 0.01. One representative experiment of three is shown. E) Schematic of the experimental evolution protocol. F-G) PCR analysis of AKAP7 at passage 1 to 10 in macrophages and L2 fibroblasts, respectively. The black arrow indicates full-length intact AKAP7, while red arrows indicate AKAP7 amplicons with deletions. H) Replication in macrophages 24 hpi (MOI=0.01) of p0 MHVAKAP7 and p10 virus populations from L2 fibroblasts and macrophages. Population 1 p-value=0.0257, Population 2 p-value=0.0058, Population 3 p-value=0.0054. Statistical significance determined by unpaired t-test. I) Replication of plaque purified p10 isolates from L2 fibroblasts (gray bars) and macrophages (black bars), in macrophages 24 hpi (MOI=0.01). J-K) PCR analysis of ORF2 in macrophages and L2 fibroblasts, respectively.

To test the impacts of distinct selective pressures on coronavirus genome composition, we used a recombinant mouse hepatitis virus (MHV) that has an inactivating H126R amino acid change in NS2, its native PDE, which is functionally complemented by insertion of the coding sequence for the cellular AKAP7 central domain PDE (AKAP7)^8^ (Figure 1A–B). The AKAP7 gene inserted into MHV is 618 nucleotides and replaces the defective 321 nucleotide ORF4, so expansion of the genome is minimal (~1%). MHV with inactive NS2 (MHV^NS2Mut^) is restricted in mouse primary bone marrow-derived macrophages (BMDMs) by OAS-RNase L (ref 18), while AKAP7 inserted into MHV (MHV^AKAP7^) compensates for the inactive NS2 and restores replication to wild-type virus levels in BMDMs and mouse liver. MHV^NS2mut^ is restricted in immortalized primary macrophages^22^, but replicates to wild-type levels in L2 mouse fibroblasts (Figure 1C), providing differential selection for courses of experimental evolution^18^. Similarly, MHV^AKAP7^ replicated to high titers whereas MHV encoding an inactive AKAP7 PDE (MHV^AKAP7mut^) was significantly restricted (Figure 1D), demonstrating that these cells have an active OAS-RNase L pathway, which we hypothesized imposes selective pressure to retain an active PDE. In contrast, MHV^AKAP7^ and MHV^AKAP7mut^ replicated to an equivalent level in L2 fibroblasts, establishing these cells as a model for MHV^AKAP7^ evolution under relaxed selection (Figure 1E).

### Deletion of AKAP7 occurs rapidly under relaxed selection

Our experimental evolution workflow uses serial passage in macrophages and L2 cells to model evolution of a horizontally acquired PDE under different conditions (Figure 1E). We plaque purified passage 0 (p0) MHV^AKAP7^ and passaged this virus in triplicate in each cell type at a multiplicity of infection (MOI) of 0.01. At each passage we titrated virus replication by plaque assay to calculate the MOI for the subsequent passage and collected total RNA for analysis of AKAP7 by PCR and Sanger sequencing. In macrophages, full-length AKAP7 was retained through ten passages (Figure 1F, Figure S1A–B), whereas in L2 cells, smaller PCR amplicons appeared by passage three or four (Figure 1G, Figure S1C–D). The smaller bands became increasingly prominent through p10, while the full length AKAP7 band faded in intensity, indicating viruses with the full-length gene were disappearing from the population (Figure 1G, Figure S1C–D). We cloned and sequenced full-length bands from macrophages and L2 cells, and smaller bands from L2 cells at each passage.

AKAP7 from both cell types contained no mutations (Supplementary File 1). The smaller bands replacing full-length AKAP7 during L2 passage featured substantial deletions (Supplementary File 1). The AKAP7 deletion variants did not undergo mutation, consistent with high fidelity coronavirus replication and revealed a lower barrier to genetic change by deletion than substitution, which has been described in SARS-CoV-2 as well^23^. Virus populations recovered at p10 from L2s exhibited significantly restricted replication relative to macrophage-passaged virus populations (Figure 1H, Figure S2A–B), demonstrating that despite the heterogeneity in AKAP7 deletion patterns between replicates, all three MHV^AKAP7^ populations lost the ability to effectively suppress OAS-RNase L.

We then plaque purified virus isolates from replicate three p10 virus populations from both cell types and used these isolates to infect macrophages and L2 cells. Consistent with the loss of AKAP7 during L2 passage, p10 plaque purified isolates from these cells exhibited reduced replication in macrophages but replicated to wild-type titers in L2 fibroblasts (Figure 1I, Figure S2C–E), with some experiments showing a growth advantage for AKAP7-deleted isolates, which warrants further study (Figure S2C–E). The rapid loss of AKAP7 under relaxed selection is consistent with an evolutionary model wherein newly encoded viral proteins viral genes must provide a near-immediate advantage for their parent gene to escape deletion.

### Inactive MHV ORF2 is retained under strong and relaxed selection

Like the loss of AKAP7 under relaxed selection, there is an *a priori* prediction that ORF2, which encodes an inactive PDE in MHV^AKAP7^, would be also rapidly lost from the MHV genome. ORF2, encoding the H126R inactivating substitution in NS2 should be dispensable in both macrophages and L2 fibroblasts. Surprisingly, PCR of ORF2 (Figure 1J–K) at each serial passage in macrophages and L2s, and sequencing of ORF2 after 10 passages, revealed no deletions novel mutations, or reversion of the H126R substitution (Supplementary File 2). Given the unexpected retention of inactive ORF2, we conducted five additional passages in both cell types to provide additional opportunity for deletion. We sequenced the macrophage-passaged viruses again at p15 to confirm there was no reversion at amino acid position 126 that might suggest ORF2 retention in these cells, even out to passage 15, was due to OAS-RNase L-mediated selection (Figure 1I–J; Supplementary File 2). This unpredicted retention of ORF2 raised the possibility that protein-coding coronavirus genes might be regularly retained under constraint distinct from protein function.

To investigate whether similar patterns of constraint exist in the wild, we analyzed ORF2 from other betacoronaviruses in the same subgenus as MHV. Rabbit coronavirus HKU14 (ref 24) has a premature stop codon mutation truncating NS2 to 43 amino acids and additional downstream stop codons in the same frame. However, the entire nucleotide sequence encoding full-length NS2 is present in 4/5 HKU14 sequences in NCBI, while the other has ~100 bp of missing sequence (Supplementary FIle 3). In contrast, ORF2 in porcine hemagglutinating encephalomyelitis virus (PHEV) appears much more evolutionarily malleable^25–28^, with various isolates containing ORF2 with premature stop codons or deletions (Supplementary File 3). These findings are broadly consistent with constraints on deletion of ORF2, with the heterogeneity in PHEV suggesting there are dispensable genomic regions.

### Long read sequencing confirms retention of ORF2 after experimental evolution

**Figure 2.**
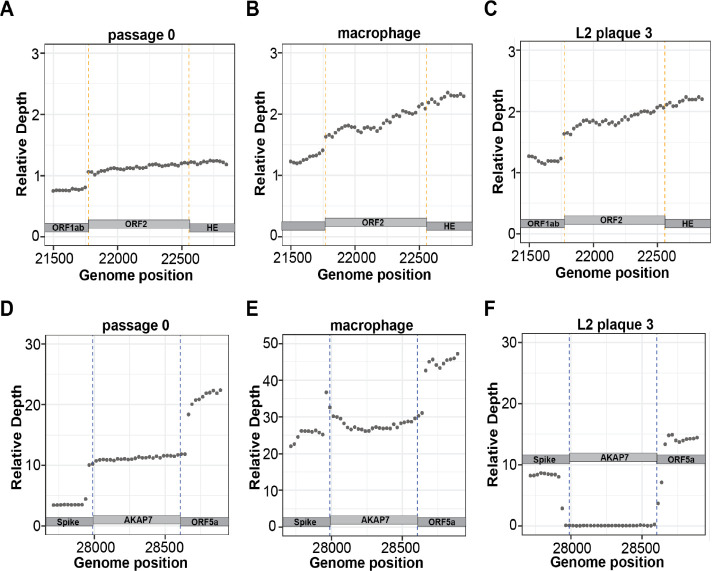
Nanopore direct cDNA sequencing shows retention of ORF2 and near-complete loss of AKAP7 following serial passage. A-C) Relative coverage depth plots of ORF2 in purified plaque isolates from passage 0 MHV^AKAP7^, and passage 10 macrophage and L2 fibroblast purified plaques. D-E) Relative coverage depth plots of AKAP7 in purified plaque isolates from passage 0 MHV^AKAP7^, and passage 10 macrophage and L2 fibroblast purified plaques.

To more extensively analyze ORF2 and AKAP7 genes after experimental evolution we performed Oxford Nanopore direct cDNA sequencing of plaque purified MHV^AKAP7^ p0, five isolates after 10 passages in L2 cells and p10 isolate from macrophage infections. PCR analysis of the plaque purified isolates showed AKAP7 was uniformly intact in macrophage-derived isolates but deleted in L2-derived plaque isolates (Figure S3A–B). Mean read length across direct cDNA Nanopore sequencing runs ranged from 1,065 to 1,528 bases and average coverage was 2,603x to 27,296x per nucleotide of the MHV^AKAP7^ genome with a mean of 8,943x. Across all sequencing runs ~70–80% of reads aligned to the MHV^AKAP7^ genome (Supplementary File 5). To quantify changes in ORF2 and AKAP7 gene content after ten passages and control for variable overall sequencing depth across runs, we normalized average coverage of ORF2 and AKAP7 to average coverage of ORF1ab for each isolate. Consistent with PCR analysis, average relative coverage of ORF2 was the same in plaque purified passage 10 isolates from macrophages and L2 fibroblasts (Figure 2A–C; Figure S3C–F), demonstrating that this genomic region is under unanticipated constraint. The average relative AKAP7 coverage for p0 and macrophage p10 were 11.2 and 27.8, respectively (Figure 2D–E). In contrast, the average relative AKAP7 coverage for L2 p10 isolates was 0.332, a 33-fold decrease from p0 consistent with PCR analysis. (Figure 2F; S3G–J).

### SARS-CoV-2 ORF8 is retained despite loss of coding capacity

Our analysis of ORF2 in MHV-like viruses suggested the constraints we identified experimentally also exist in virus populations in the wild. However, the strength of this analysis was limited by the relative paucity of genomic data available for animal viruses. In contrast, the SARS-CoV-2 dataset in the Global Initiative on Sharing All Influenza Data (GISAID) database contains more than 16 million genomes, offering an unprecedented window into viral evolution. Analogous to the inactivation of MHV ORF2 in our system, SARS-CoV-2 ORF8 has acquired premature stop codons in numerous globally successful lineages, including B.1.1.7, XBB.1, XBB.1.5, XBB.1.9, and XBB.1.16 (ref 21), and a mutation in the ORF8 transcriptional regulatory sequence (TRS) in the BA.5 lineage that substantially reduces ORF8 subgenomic RNA and protein synthesis^29^. ORF8 has been of interest since early in the SARS-CoV-2 pandemic due to a small deletion that emerged in its SARS-CoV ortholog in 2003 ^30^, an early cluster of infections in Singapore involving an ORF8 deletion variant^31,32^, as well as debate over its role during SARS-CoV-2 infection^6,32–39^. Despite the intense focus on ORF8 function and loss of full-length protein synthesis, very little attention has been devoted to the evolution of the gene itself.

**Figure 3.**
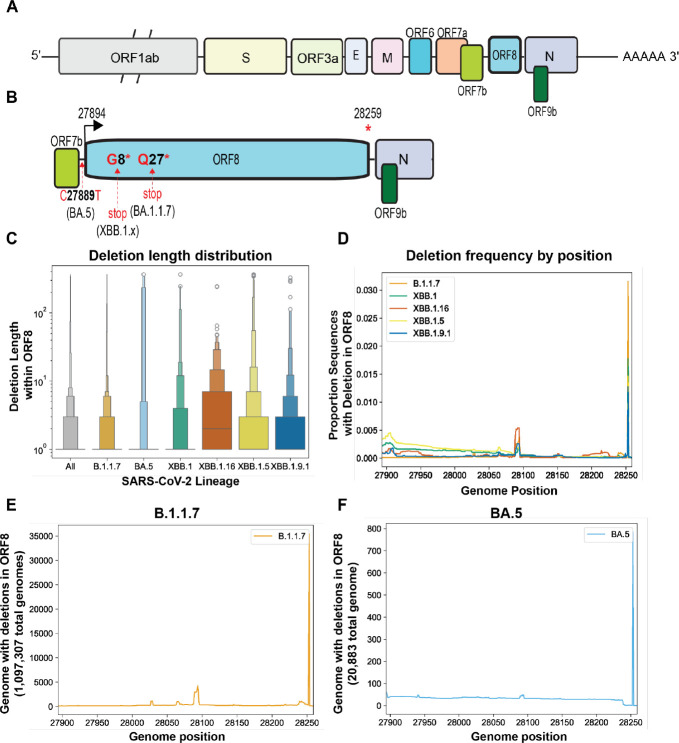
ORF8 is retained in SARS-CoV-2 lineages despite mutations that disrupt protein coding. A) Schematic of SARS-CoV-2 genome. B) Schematic (with ORF8 expanded, not to scale) of ORF8, with mutations resulting in loss of subgenomic mRNA synthesis (BA.5) or an early stop codon (B.1.1.7 and XBB.1.x) indicated. C) Deletion length distribution - this displays the size of all deletions in ORF8 in each lineage from 0 to >100 nucleotides. D-F) Proportion of genomes from lineages with a deletion at each position in SARS-CoV-2 ORF8.

We downloaded all sequences assigned to the B.1.1.7, BA.5, XBB.1, XBB.1.5, XBB.1.9, and XBB.1.16 lineages from GISAID and applied a cutoff date to exclude any samples without metadata or with metadata suggesting they are incorrectly assigned (Figure S4). For each lineage, we further defined a start-date for circulation and calculated the date at which 50% and 90% of sequences assigned to this lineage were collected, as of November 29, 2023. We further excluded any sequences with any ambiguous nucleotides in the ORF8 gene to eliminate confounding of our analysis by poor-quality sequences, which might obscure *bona fide* deletions. We calculated a deletion length distribution of ORF8 in B.1.1.7 (1,097,307 sequences), BA.5 (20,833 sequences), XBB.1 (22,031 sequences), XBB.1.5 (166,068 sequences), XBB.1.9.1 (19.990 sequences), and XBB.1.16 (19,119 sequences) (Figure 3C). Across all lineages 96% of deletions were smaller than ten nucleotides, ranging from a high of 98.15% of deletions smaller than ten nucleotides for B.1.1.7 to a low of 86.6% of deletions smaller than ten nucleotides for XBB.1.16. (Figure 3C, Supplementary File 5). We then analyzed all sequences in the dataset to determine whether any regions of ORF8 were particularly deletion-tolerant or resistant. While XBB.1.5 ORF8 deletions were enriched in the 5’ half of the gene (Figure 3D, S5B), deletions were uniformly rare at all positions across the other lineages we analyzed (Figure 3D–F, S5A–D) except for a single site at the 3’ end that we speculate may impact sgRNA synthesis of downstream ORFs N and 9b.

**Figure 4.**
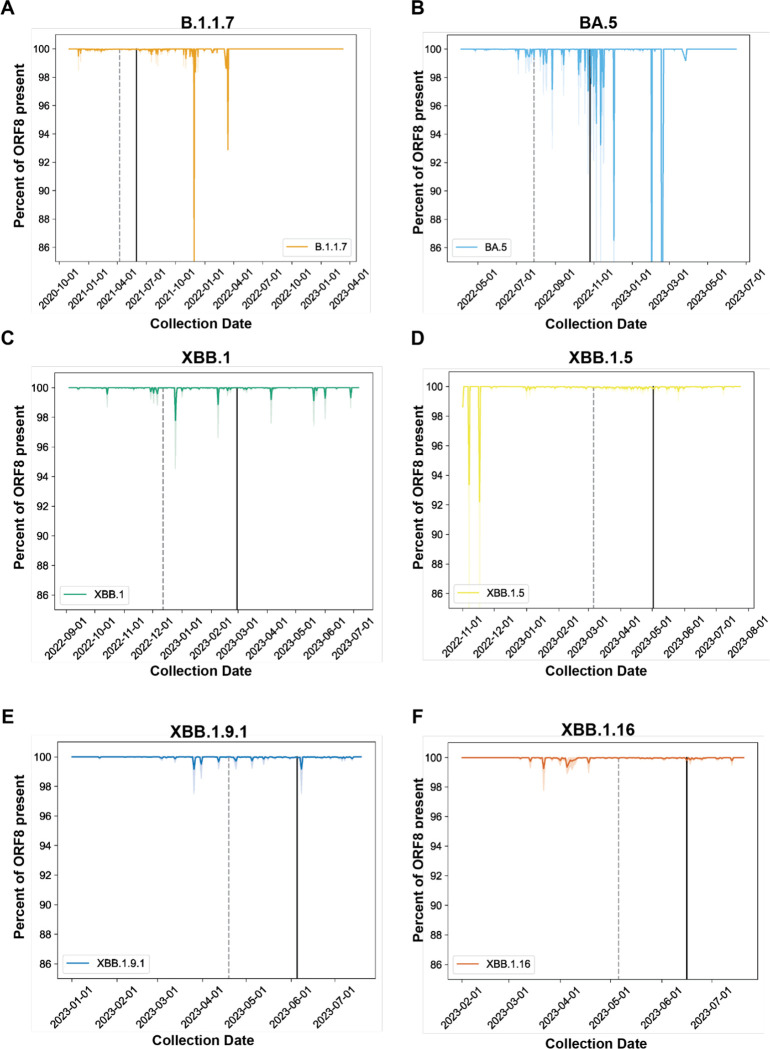
SARS-CoV-2 ORF8 is recurrently retained following acquisition of an early stop codon. A-F) Line plots of percent nucleotide content for the indicated SARS-CoV-2 lineages over time throughout the course of their circulation in humans. Dark lines are the mean percentage for all sequences collected on that date, and faded lines are the 95% confidence interval. Dashed vertical line is the date by which 50% of sequence assigned to the designated lineage were collected, and the solid line is the 90% cutoff.

To determine whether ORF8 in these lineages degraded over time, we calculated the percent of ORF8 gene content in these lineages, sorted by collection date, and plotted these values for all genomes of all six lineages (Figure 4A–F). To determine how the appearance of ORF8 deletions in these lineages related to their epidemiological rise and fall, we calculated the dates at which 50% (dashed vertical line) and 90% (solid vertical line) of sequences assigned to each lineage had been collected. For all lineages, genomes with deletions were rare outside this window, generally occurring early after the appearance of a lineage and going extinct during lineage growth or emerging very late when the lineage was already in steep decline.

## Discussion

Findings from this study challenge a simple evolutionary model wherein the fate of protein coding genes hinges only on protein function. The sustained, unexpected retention of ORF2 in both macrophages and L2 fibroblasts through fifteen passages suggests a complex interplay of selective pressure acting on viral coding sequence. In L2s the PDE activity of ORF2 is dispensable, and the stability of the H126R substitution during experimental evolution in macrophages is consistent with AKAP7 fully counteracting OAS-RNase L activity. While it is formally possible that an unknown secondary role for the ORF2-encoded protein NS2 antagonizes innate immunity, no such function has been identified and the protein contains no identifiable domains other than the PDE.

The difference in genomic location between ORF2 and AKAP7 may be relevant to their respective fates. AKAP7 is located upstream of ORF5a, which encodes an accessory gene that promotes resistance to interferon signaling^41^, whereas ORF2 immediately precedes the hemagglutininesterase (HE) and spike structural genes. HE subgenomic mRNA is not expressed from the MHV A59 genome^42,43^, so it is unlikely that the selection against ORF2 deletion relates to transcription of the immediate downstream gene.

Under a conventional model of selection favoring genome streamlining, viruses with ORF8 deleted would quickly dominate populations soon after the potential to encode full-length protein was lost. The exact nature of the evolutionary constraint on MHV ORF2 and SARS-CoV-2 ORF8 is not yet clear. A leading possibility is that at least some coronavirus genes contain regulatory functions in addition to their protein coding capacity that tune the transcription of other genes. MHV ORF2 is upstream of spike, albeit with the defective HE gene intervening, and SARS-CoV-2 ORF8 immediately precedes nucleocapsid, which has multiple functions key to promoting viral fitness^44^. If accessory genes promote transcription of spike, nucleocapsid, and other subgenomic RNAs, loss of such genes might reduce viral fitness and lineages containing such deletions would go extinct. Increased transcription of subgenomic RNAs encoding innate immune antagonists has been linked to enhanced suppression of innate immunity in major SARS-CoV-2 variants of concern^45^, which is consistent with our hypothesis and analysis of SARS-CoV-2 ORF8 retention.

The loss of ORF2 in HKU1 and the observation of some ORF8 deletion SARS-CoV-2 viruses indicates that patterns of constraint may be subtle or absent in some genomic regions. In the case of SARS-CoV-2, the apparent decreased fitness of ORF8-deletion viruses might be balanced by adaptations that compensate for loss of ORF8, affording fitness benefits of genome compression while shedding the deleterious impact of losing the gene. Continued surveillance of SARS-CoV-2 might reveal such variants over and provide more insight into fundamental factors driving coronavirus evolution.

Identifying additional patterns of evolutionary constraint may reveal other sequence features impacting viral fitness. Patterns of selection can be prioritized for mechanistic studies using reverse genetic strategies established for MHV and SARS-CoV-2, opening a bridge between experimentation and computational analysis of viral evolution. Experimentally dissecting fitness impacts will benefit from future competition studies. For example, the loss of ORF2 in HKU1 results in viable viruses and fitness defects of MHV^ΔORF2^ might appear only in direct competition with wild-type viruses and not in parallel courses of experimental evolution. Overall, this work demonstrates the power of merging experimental approaches with computational analysis of massive datasets to identify patterns of constraint influencing the evolution of emerging and pandemic viruses.

## Materials and Methods

### Cells and Viruses

Immortalized primary macrophages were provided by Sunny Shin^22^, 17-Clone 1 and L2 cells were provided by Susan Weiss. Macrophages were cultured in RPMI-1640 supplemented with 10% FBS, 1% L-glutamine, and 1% Penicillin/Streptomycin, while 17-Clone 1 and L2 cells were cultured in DMEM supplemented the same way.

All viruses used in this study were provided by Susan Weiss at the University of Pennsylvania. Wild-type MHV and MHV^NS2mut^ were previously described^18^ as were MHV^AKAP7^ and MHV^AKAP7mut8^. Viruses were received as seed aliquots and 100 μl of each virus was added to one T75 flask containing a confluent monolayer of 17-Clone 1 cells in 2 ml volume. Flasks were incubated for one hour at 37° C, after which 10 ml of fresh media was added. Once widespread cytopathic effect was present (18–24 hours post-infection) the flasks were put through three freeze-thaw cycles and the supernatant was clarified via centrifugation and aliquoted for later use.

### Virus infections and plaque assays

One day after seeding in 12-well plates macrophages or L2 cells were infected at an MOI of 0.01 in 200 μl total volume and incubated for one hour at 37 ° C with rocking every 15 minutes. After one hour cells were washed 3 times with PBS and 1 ml of fresh media (2% FBS) was added to each well. 24 hours post-infection 300 μl of supernatant was collected and stored at −80° C. We also harvested total RNA for additional analyses (described below). Initial infections, all serial passages, and endpoint replication comparisons were done at an MOI of 0.01. Viral titers were quantified by plaque assay on confluent L2 cell monolayers. Supernatants collected 24 hours post-infection were serially diluted 1:10 to 10^−8^, inoculated onto L2 monolayers and incubated for 1 hour at 37 ° C with rocking every 15 minutes. After one hour 3 mL of semi-solid agarose overlay was added to each well and the plates returned to 37 ° C for 24 hours. 24 hours post-infection the agarose overlay was removed and the monolayers were washed with PBS and stained with 0.5% crystal violet plus 20% methanol. The stain was washed off with water and plaques counted and recorded for analysis.

### Statistical testing

Statistical testing of viral titers (Figure 1C–D, H-I; Figure S2) was conducted in GraphPad Prism 10, via unpaired t-test.

### RNA extraction and cDNA synthesis

Total RNA was harvested 24 hours post-infection on each serial passage with the Zymo Research Quick-RNA Miniprep Kit (Cat. #R1057) following the manufacturer’s protocol. To increase yield we eluted the RNA in a reduced volume of 25 μl nuclease-free water. RNA quantification and quality assessment was conducted using a Synergy HT BioTek plate reader. cDNA was synthesized using the Thermo Scientific Maxima First Strand cDNA Synthesis Kit with dsDNase (Lot. 2664799) with 1 μg of input RNA.

### PCR and Sanger sequencing

At each passage the AKAP7 central domain and ORF2 genes from MHV^AKAP7^ were analyzed by PCR. We used Phusion Flash HiFi Master Mix (Cat # F548S ) in 20 μl/reaction with 2 μl of cDNA template. Thirty cycles of PCR were performed. Cycling conditions were: **1)** Denaturation, 98°C for 10 seconds **2) a)** Denaturation (98°C for 2 seconds) **b)** Annealing (63°C for 10 seconds) **c)** Extension (72°C for 30 seconds) **d)** Final extension (72°C for 2 minutes) **3)** Hold (4°C).

PCR products were visualized by agarose gel (1%) electrophoresis then extracted and purified using Zymoclean Gel DNA Recovery Kit (Zymo Research Lot. #213587) following manufacturer protocols. DNA was eluted in 10 μl. Purified PCR products were prepared for Sanger Sequencing by TOPO cloning using the Invitrogen TA Cloning^™^ Kit, with pCR^™^2.1 Vector (Cat. #K202020) following manufacturer protocols. Plasmid DNA was extracted using the Zippy Plasmid Miniprep Kit (Zymo Research Cat. # 11–308) following manufacturer protocol with elution 75 μl nuclease-free water. Plasmid DNA was quantified with a Synergy HT BioTek plate reader and sent for sequencing to the University of Utah DNA sequencing core or Genewiz.

### Sequencing primers

**Table:** 

Primers	Forward	Reverse
AKAP7	5’-**ATTGCTACCTGGCCCCG**-3’	5’-**CTAGGGTCTTAGGCCCAAATG**-3’
MHV ORF2	5’-**ATGGCCTTTGCTGACAAGCCTAAT**-3’	5’-**TCAACACATACAACCCTTCATTCT**-3’
M13	5’-**GTAAAACGACGGCCAG**-3’	5’-**CAGGAAACAGCTATGAC**-3’

#### Sequence analysis

Sequence files were imported into Geneious and aligned to either the AKAP7 or ORF2 coding sequences using the MAFFT Geneious plug-in ^46^. HKU1, HKU14, and PHEV multiple sequence alignments were likewise generated using the MAFFT Geneious plug-in with default parameters.

#### Oxford Nanopore direct cDNA sequencing

1 mL of media containing a plaque isolate was used to infect 2× 10 cm dishes of desired cell type (macrophages or L2). 18–20 hours post-infection total RNA was collected using Zymo Research Quick-RNA Miniprep Kit (Cat. #R1057). The same lysis buffer (1 ml) was used on both dishes/per infection to maximize yield. Polyadenylated RNA was extracted using the Invitrogen Poly(A)Purist MAG Kit (Cat # AM1922) following the manufacturer protocol. 100 ng PolyA+ RNA was prepared for sequencing using the Direct cDNA Sequencing Kit SQK-DCS109 from Oxford Nanopore Technologies, according to the manufacturer’s instructions. The prepared cDNA libraries were sequenced using the MinION Mk1C with FLO-MIN106D flow cells, version 9.4.1. Fastq files were generated from fast5 files using the high-accuracy model of Guppy basecaller, version 6.5.7 with the following parameters hac_guppy -c dna_r9.4.1_450bps_hac.cfg -x auto --compress_fastq.

#### Direct cDNA sequencing coverage analysis

Fastq files from Nanopore sequencing runs were individually aligned to the MHV^AKAP7^ (passage 0) reference genome. Alignments were performed using minimap2 (v.2.23) and subsequently sorted and indexed with samtools (v.1.16). Coverage at every position across the genome for each sample was calculated using the bedtools (v.2.26.0) genomecov command. Coverage was then normalized for each sample to the average coverage across ORF1ab (nucleotide positions 211–21746 in the reference genome), which contains the non-amplified reading frame, allowing for comparison across sequencing runs and samples. A rolling average of this normalized read depth was calculated across the genome in 25 bp windows and plotted with ggplot2 in R. For AKAP7 and ORF2 analysis, rolling averages were recalculated for the region containing the gene sequence ± 300 bp of flanking sequence on either side and plotted identically to the genome wide coverage plots. Fastq sequencing files are available in the Sequence Read Archive at BioProject ID PRJNA1031673.

### SARS-CoV-2 ORF8 Deletion Analysis

All methods and code used to conduct this analysis are available at https://github.com/niemasd/SC2-Deletion-Analysis.

## Supplementary Material

Supplement 1

Supplement 2
