# Supplementary material for: Hidden evolutionary constraints dictate the retention of coronavirus accessory genes
Source: bioRxiv. 2024 Oct 9:2023.10.12.561935. Originally published 2023 Oct 14. Preprint. [Version 3] doi: 10.1101/2023.10.12.561935 (PMC10592793; doi:10.1101/2023.10.12.561935)
Supplement: Supplement 3 [file NIHPP2023.10.12.561935v3-supplement-3.pdf]

bioRxiv preprint doi: https://doi.org/10.1101/2023.10.12.561935; this version posted October 9, 2024. The copyright holder for this preprint (which was not certified by peer review) is the author/funder, who has granted bioRxiv a license to display the preprint in perpetuity. It is made available under aCC-BY 4.0 International license.

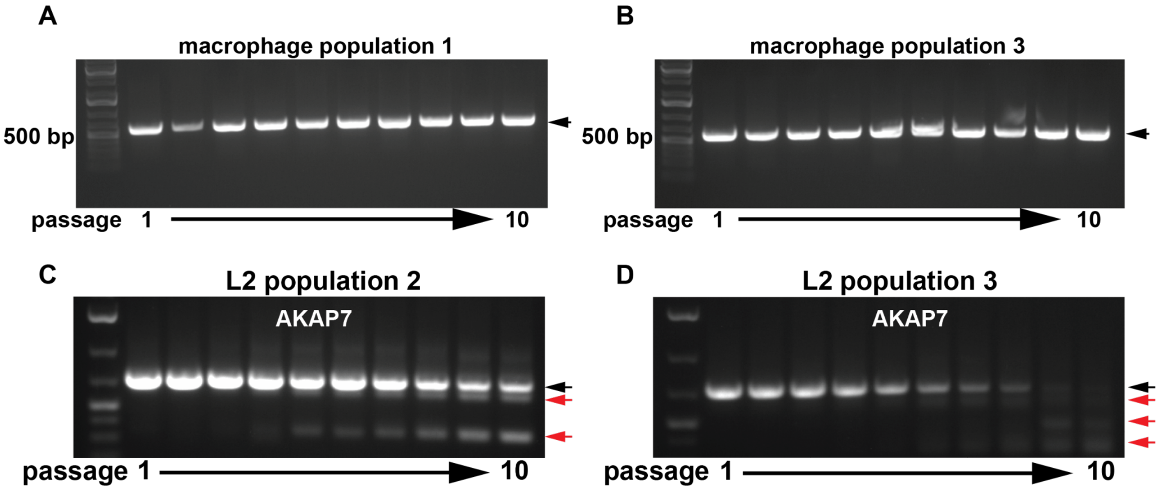

FIgure S1. PCR analysis of AKAP7 during serial passage, related to Figure 1. A and B) AKAP7 PCR of passages 1 to 10 in macrophages. C and D) AKAP7 PCR of passages 1 to 10 in L2 experimental evolution replicate 2 and 3. Black arrows indicate full-length AKAP7, and red arrows indicate AKAP7 amplicons containing deletions.

bioRxiv preprint doi: https://doi.org/10.1101/2023.10.12.561935; this version posted October 9, 2024. The copyright holder for this preprint (which was not certified by peer review) is the author/funder, who has granted bioRxiv a license to display the preprint in perpetuity. It is made available under aCC-BY 4.0 International license.

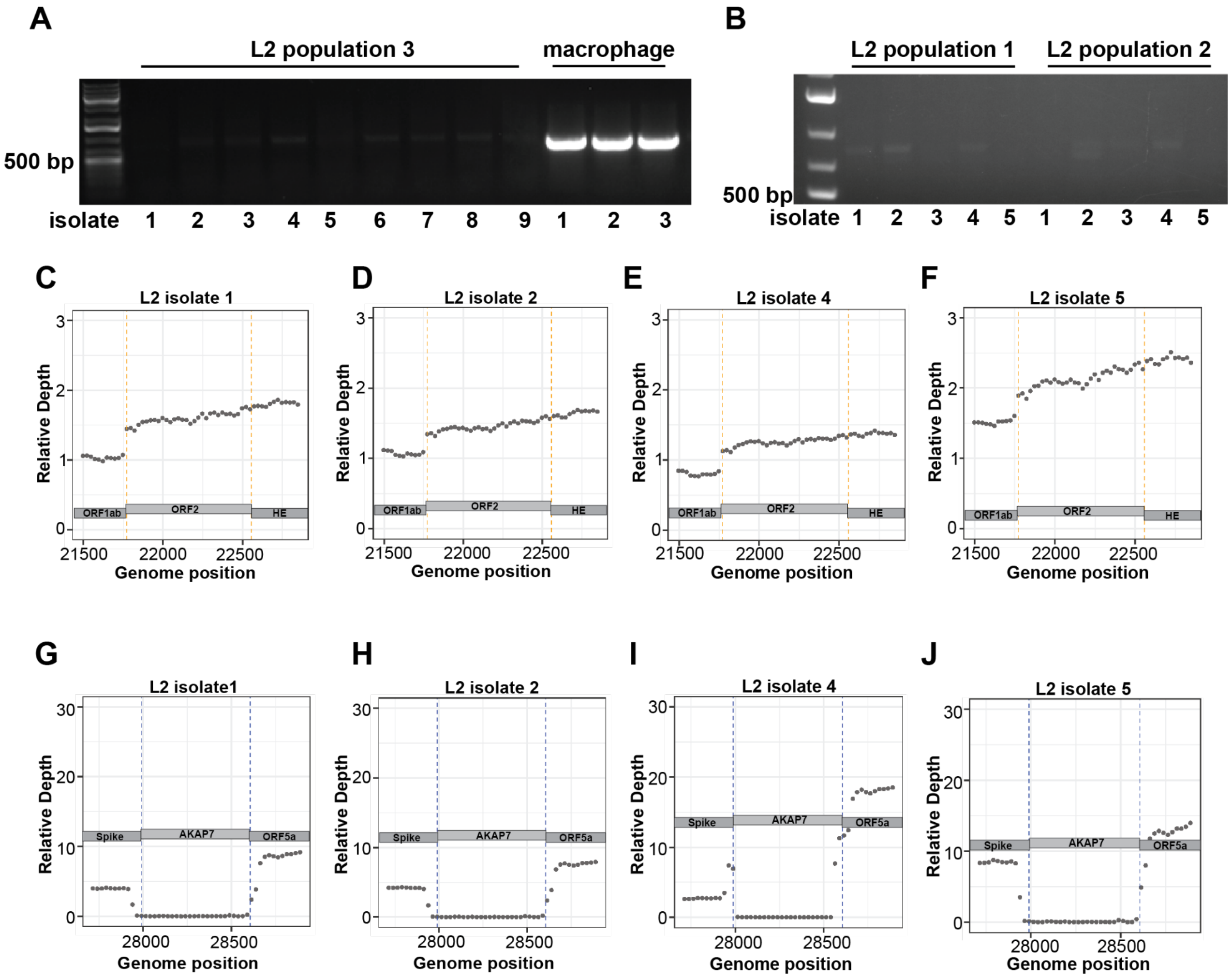

Figure S2. Direct cDNA sequencing shows loss of AKAP7 and retention of ORF2 during serial passage, related to Figure 2. A-B) PCR analysis of AKAP7 in plaque purified p10 MHV^{AKAP7} isolates**.** C-F) Relative coverage depth plots of ORF in purified plaque isolates from p10 L2 fibroblast purified plaque isolates. G-J) Relative coverage depth plots of AKAP7 in purified plaque isolates from p10 L2 fibroblast purified plaque isolates.

bioRxiv preprint doi: https://doi.org/10.1101/2023.10.12.561935; this version posted October 9, 2024. The copyright holder for this preprint (which was not certified by peer review) is the author/funder, who has granted bioRxiv a license to display the preprint in perpetuity. It is made available under aCC-BY 4.0 International license.

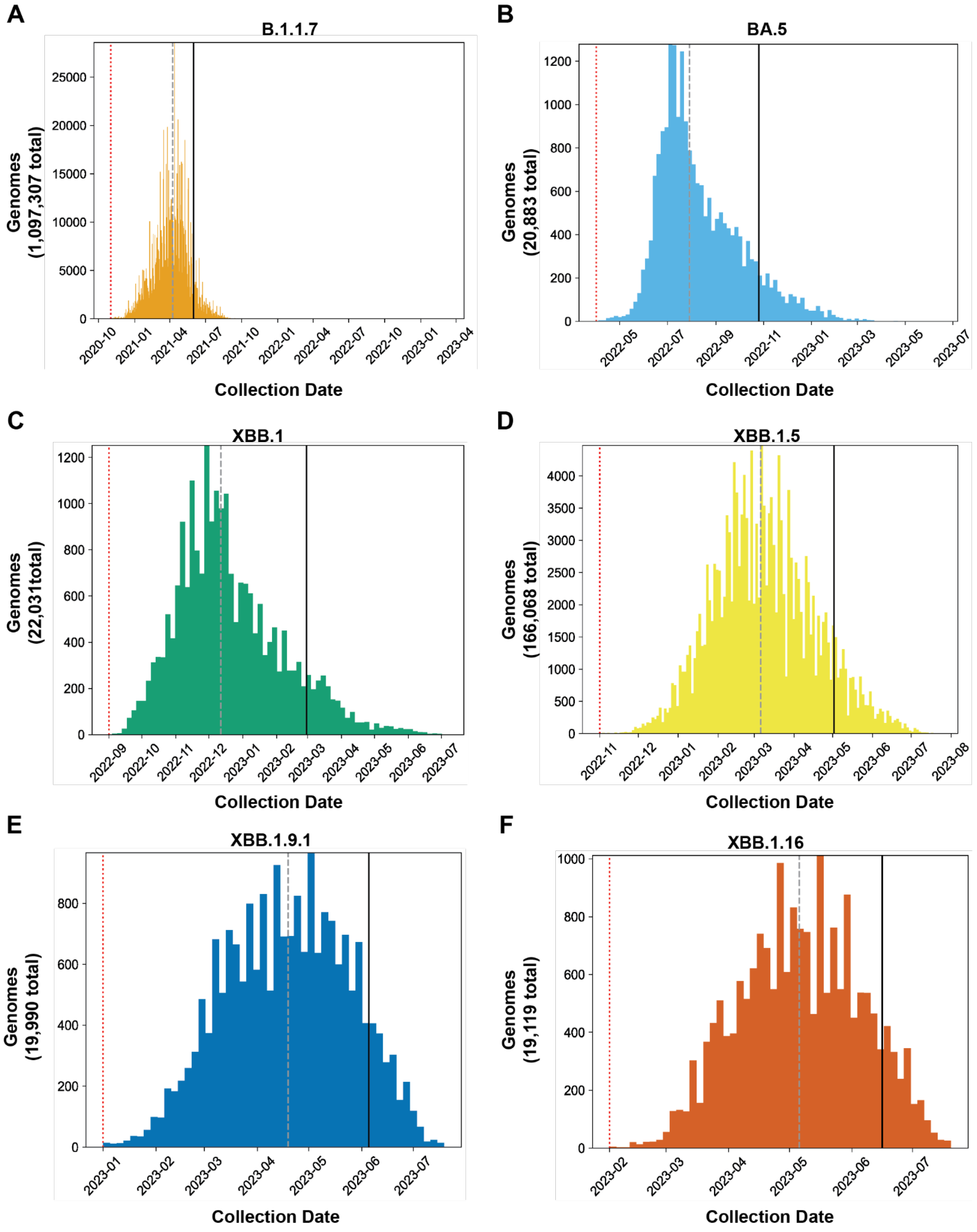

Figure S3. Histogram of ORF8 premature-stop codon-containing SARS-CoV-2 lineage genomes plotted by sampling date, related to Figures 4 and 5. A-E) Histograms of SARS-CoV-2 lineages we sampled that have premature stop codons in ORF8. The x-axis on each plot is the collection date and the y-axis indicates the number of genomes collected on each date. The red dotted line indicates the start-date of the lineage, determined by identifying the date when sequences reported from each lineage began to increase. The dashed line is the date by which 50% of all sequences assigned to this lineage were collected and the solid vertical line the date by 90% had been collected (as of 11/29/2023).

bioRxiv preprint doi: https://doi.org/10.1101/2023.10.12.561935; this version posted October 9, 2024. The copyright holder for this preprint (which was not certified by peer review) is the author/funder, who has granted bioRxiv a license to display the preprint in perpetuity. It is made available under aCC-BY 4.0 International license.

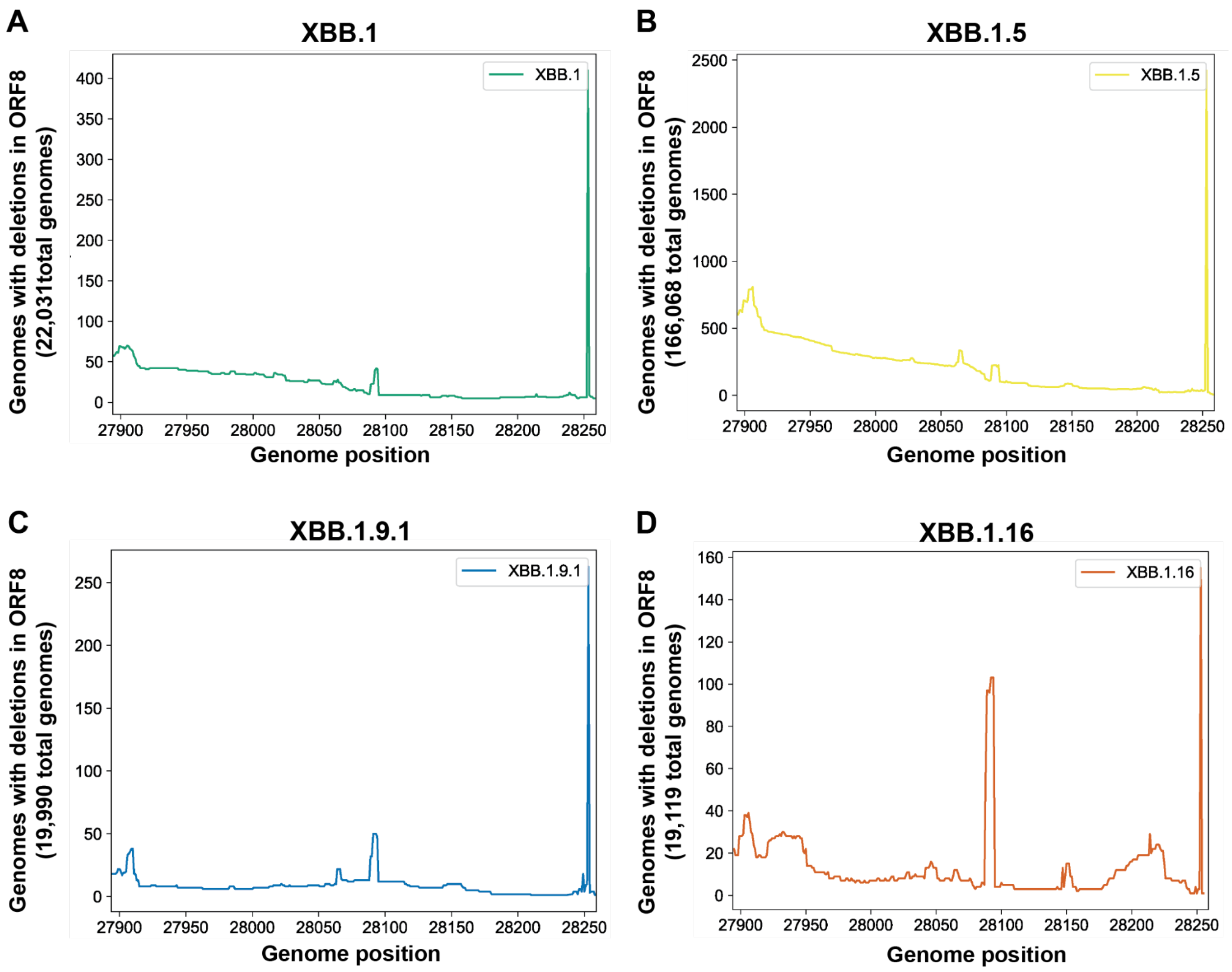

Figure S4. Deletions are not consistently enriched in discrete regions of SARS-CoV-2 ORF8, related to Figure 5. A-D) Plots depicting the number of sequences sampled on each date with deletions at each position within SARS-CoV-2 ORF8 for the indicated lineage.

bioRxiv preprint doi: https://doi.org/10.1101/2023.10.12.561935; this version posted October 9, 2024. The copyright holder for this preprint (which was not certified by peer review) is the author/funder, who has granted bioRxiv a license to display the preprint in perpetuity. It is made available under aCC-BY 4.0 International license.

	<10 nucleotides	10-100 nucleotides	>100 nucleotides
**All lineages**	97.38%	1.94%	0.68%
**B.1.1.7**	98.16%	1.36%	0.49%
**BA.5**	95.51%	0.79%	3.70%
**XBB.1**	93.27%	5.0%	1.73%
**XBB.1.16**	86.6%	12.92%	0.48%
**XBB.1.5**	90.67%	7.34%	1.98%
**XBB.1.9.1**	92.73%	5.26%	2.01%

Table S3. SARS-CoV-2 ORF8 deletion length distribution raw data, related to Figure 4C. This table contains raw data of deletion lengths in SARS-CoV-2 ORF8, specifically the percentage of deletions >10 nucleotides, 10-100 nucleotides, ad >100 nucleotides for all lineages in aggregate, and broken out into individual lineages.
