# Supplementary material for: Hidden evolutionary constraints dictate the retention of coronavirus accessory genes
Source: bioRxiv. 2024 Mar 19:2023.10.12.561935. Originally published 2023 Oct 14. Preprint. [Version 2] doi: 10.1101/2023.10.12.561935 (PMC10592793; doi:10.1101/2023.10.12.561935)
Supplement: Supplementary file 1 [file media-1.pdf]

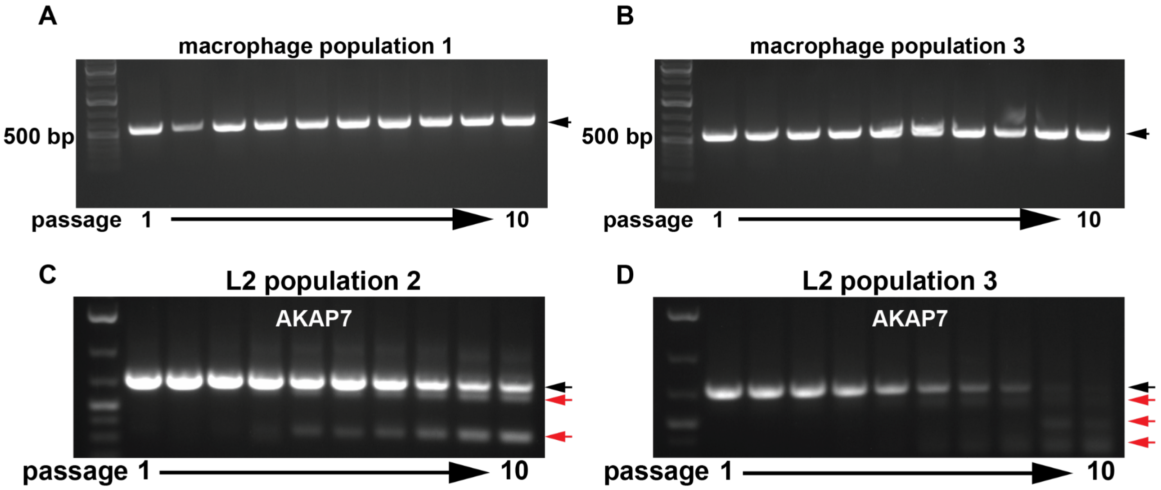

FIgure S1. PCR analysis of AKAP7 during serial passage. A and B) AKAP7 PCR of passages 1 to 10 in macrophages. C and D) AKAP7 PCR of passages 1 to 10 in L2 experimental evolution replicate 2 and 3. Black arrows indicate full-length AKAP7, and red arrows indicate AKAP7 amplicons containing deletions.

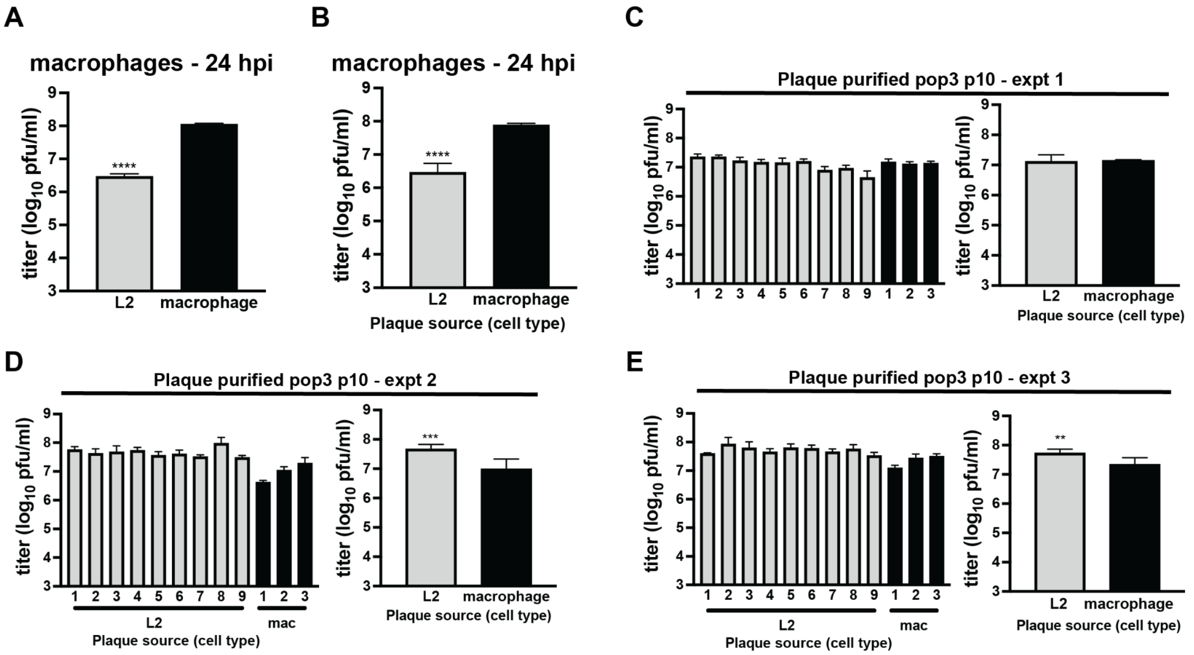

Figure S2. Replication of passage 10 viruses. A) Mean replication in macrophages of p10 virus populations passaged in L2 fibroblasts or macrophages ($p<0.0001$). B) Mean replication in macrophages of plaque-purified p10 viruses from passage in L2 fibroblasts or macrophages ($p<0.0001$) C-E) Replication of plaque purified p10 isolates in L2 cells, with all three experiments shown due to heterogeneity in results. Replication of individual plaque-purified isolates (left graph) and mean plaque-purified (right graph) are shown. p=.8307 (C) p=0.0007 (D), p=0.0044 (E). Panels (A) and (B) are representative of three independent experiments.

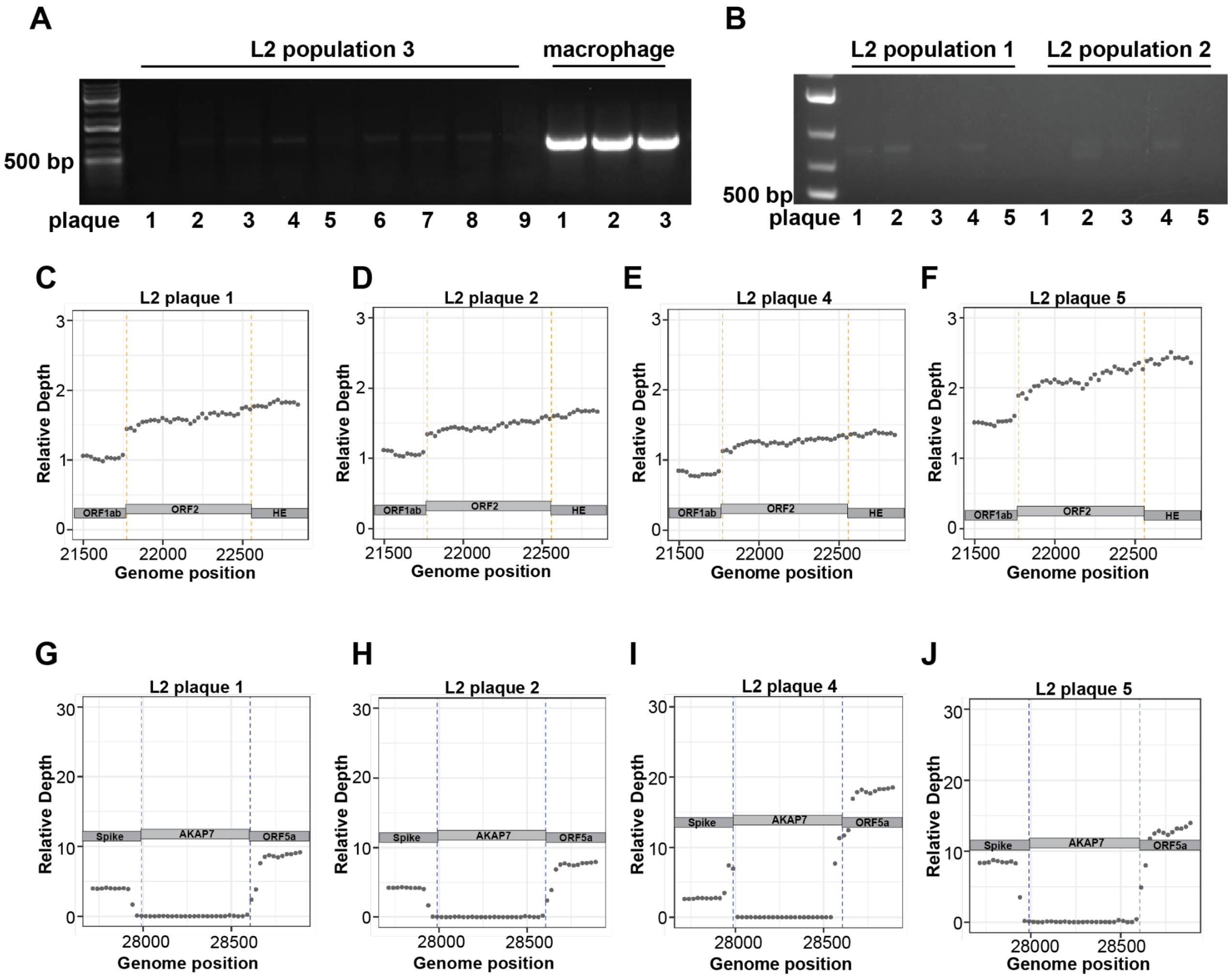

Figure S3. Direct cDNA sequencing shows loss of AKAP7 and retention of ORF2 during serial passage. A-B) PCR analysis of AKAP7 in plaque purified passage ten MHV^{AKAP7} isolates**.** C-F) Relative coverage depth plots of ORF in purified plaque isolates from passage 10 L2 fibroblast purified plaque isolates. G-J) Relative coverage depth plots of AKAP7 in purified plaque isolates from passage 10 L2 fibroblast purified plaque isolates.

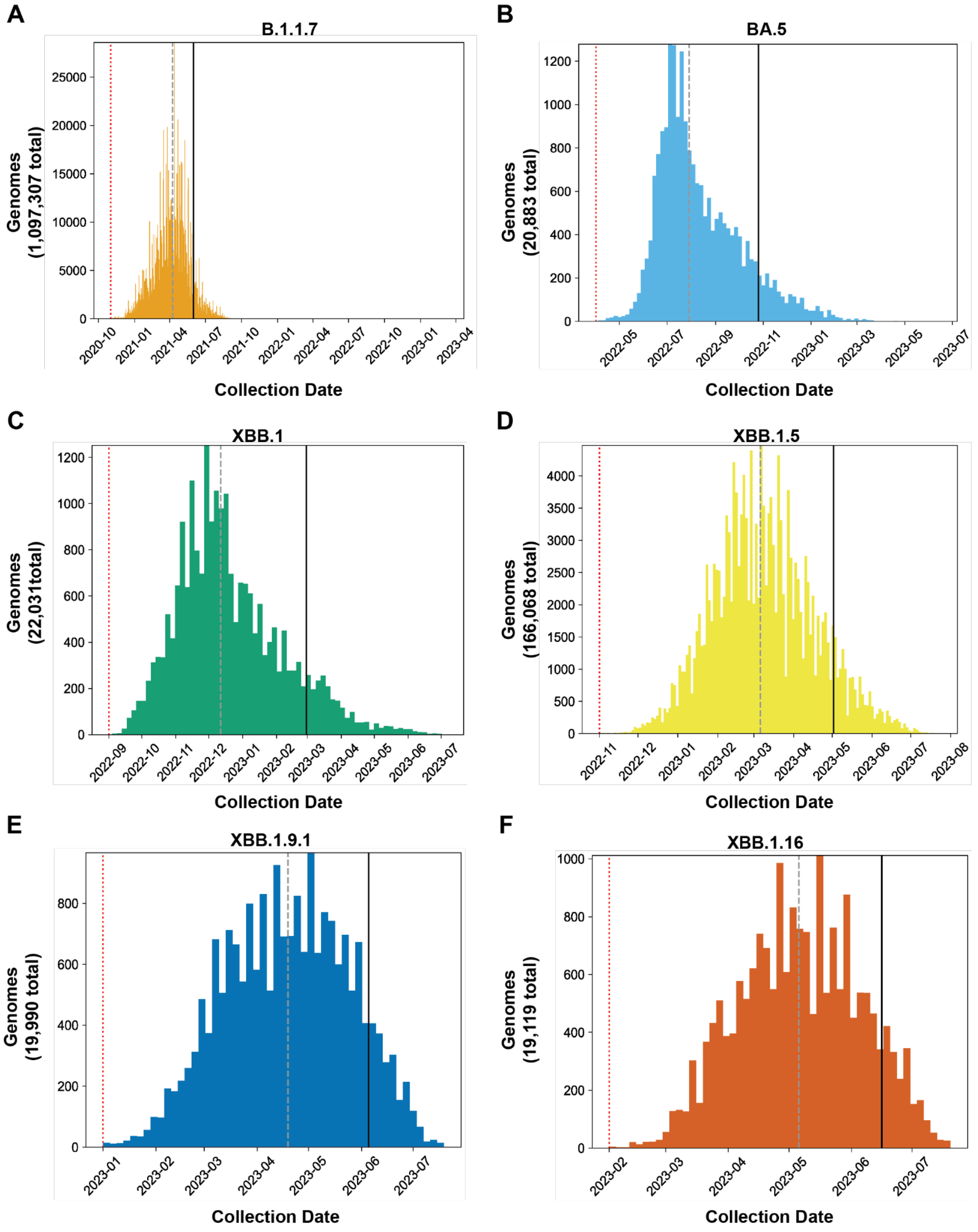

Figure S4. Histogram of ORF8 premature-stop codon-containing SARS-CoV-2 lineage genomes plotted by sampling date. A-E) Histograms of SARS-CoV-2 lineages we sampled that have premature stop codons in ORF8. The x-axis on each plot is the collection date and the y-axis indicates the number of genomes collected on each date. The red dotted line indicates the start-date of the lineage, determined by identifying the date when sequences reported from each lineage began to increase. The dashed line is the date by which 50% of all sequences assigned to this lineage were collected and the solid vertical line the date by 90% had been collected (as of 11/29/2023).

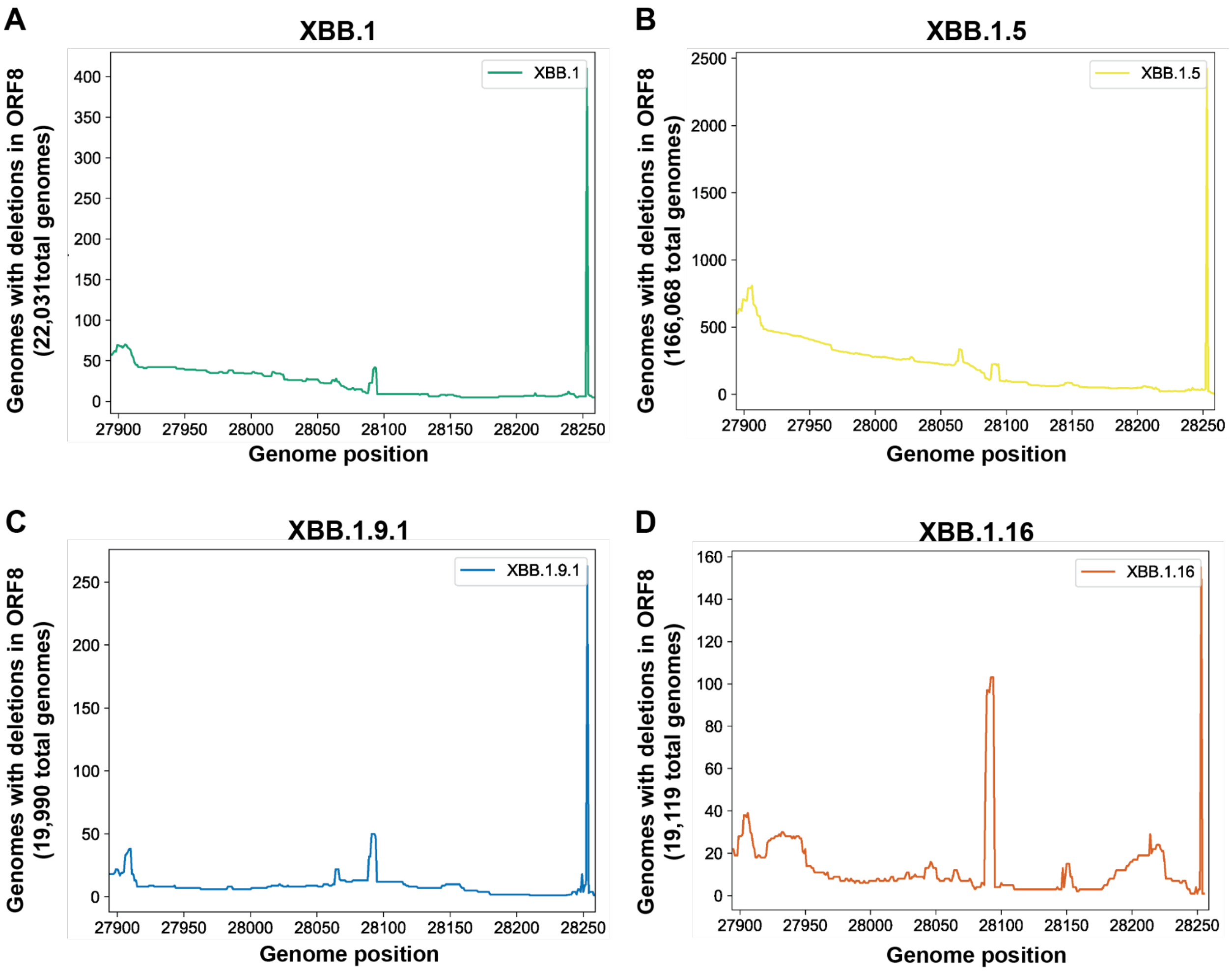

Figure S5. Deletions are not consistently enriched in discrete regions of SARS-CoV-2 ORF8 A-D) Plots depicting the number of sequences sampled on each date with deletions at each position within ORF8 for the indicated lineage.
