# Supplementary material for: Hidden evolutionary constraints dictate the retention of coronavirus accessory genes
Source: bioRxiv. 2023 Oct 14:2023.10.12.561935. Preprint. [Version 1] doi: 10.1101/2023.10.12.561935 (PMC10592793; doi:10.1101/2023.10.12.561935)
Supplement: Supplementary file 1 [file NIHPP2023.10.12.561935v1-supplement-2.pdf]

bioRxiv preprint doi: https://doi.org/10.1101/2023.10.12.561935; this version posted October 14, 2023. The copyright holder for this preprint (which was not certified by peer review) is the author/funder, who has granted bioRxiv a license to display the preprint in perpetuity. It is made available under aCC-BY 4.0 International license.

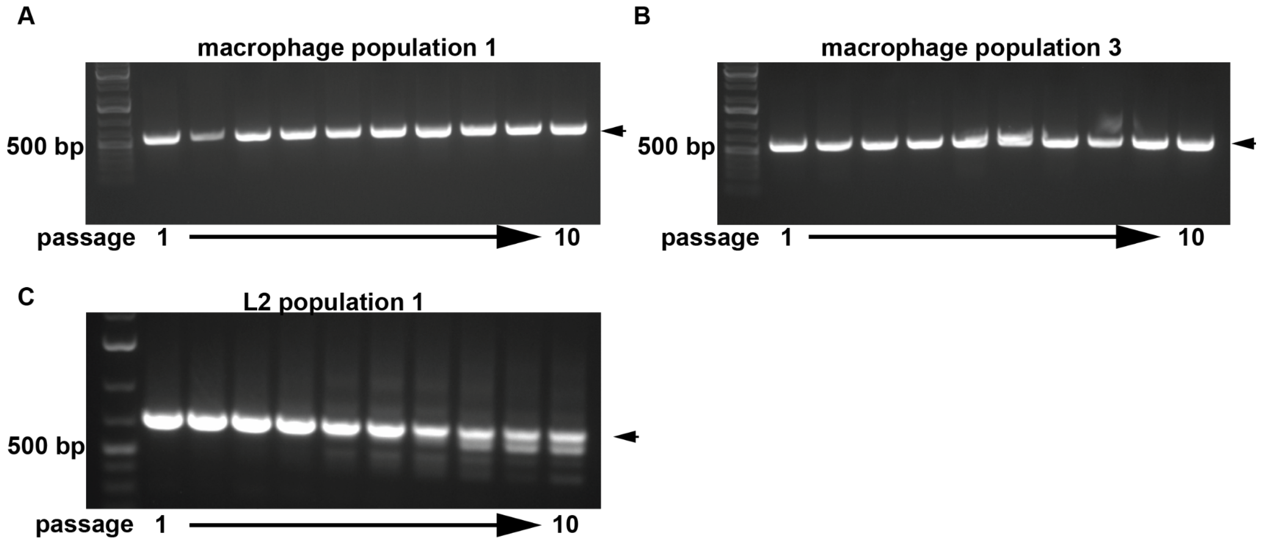

Figure S1. PCR analysis of AKAP7 during serial passage. A and B) AKAP7 PCR of passages 1 to 10 in macrophages. C) AKAP7 PCR of passages 1 to 10 in L2 experimental evolution replicate 1.

bioRxiv preprint doi: https://doi.org/10.1101/2023.10.12.561935; this version posted October 14, 2023. The copyright holder for this preprint (which was not certified by peer review) is the author/funder, who has granted bioRxiv a license to display the preprint in perpetuity. It is made available under aCC-BY 4.0 International license.

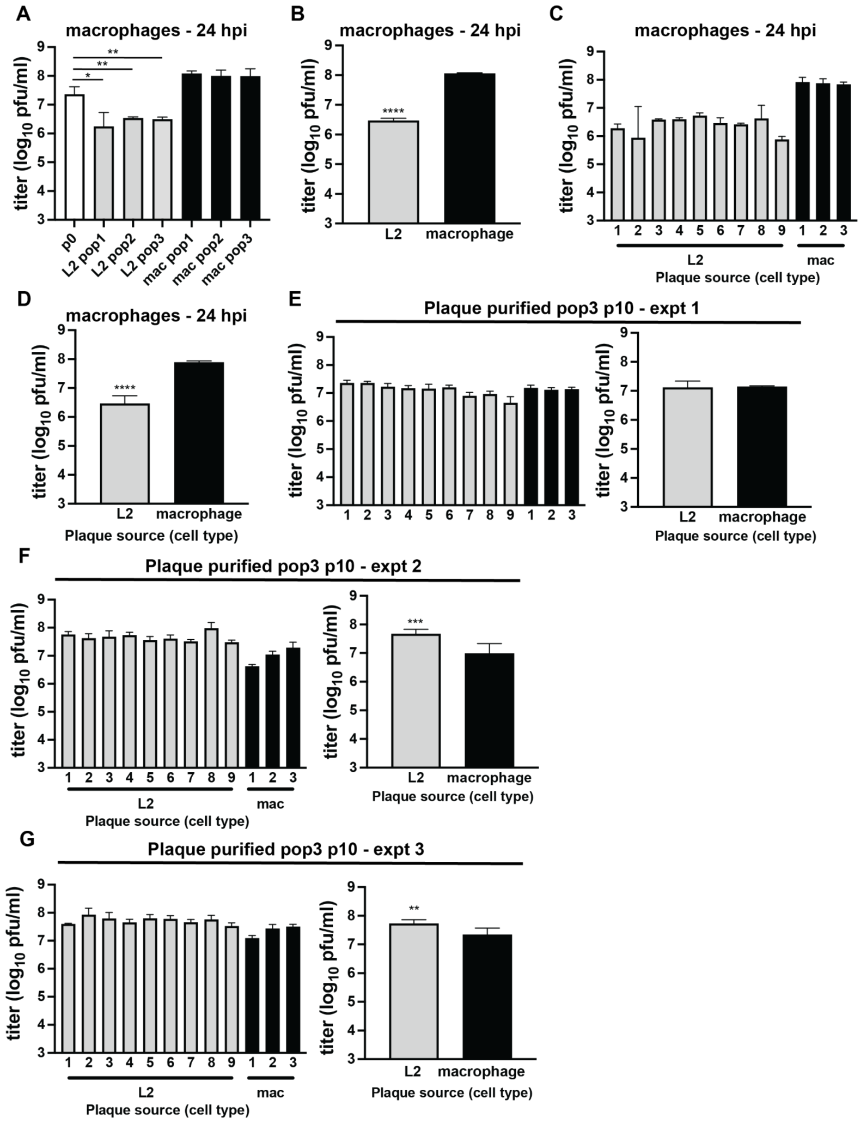

Figure S2. Replication of passage 10 viruses. A) Replication of passage 0 MHVAKAP7 and viral populations recovered from L2 and macrophage passage 10, 24 hours post-infection at MOI=0.01. p=0.0257, 0.0058, 0.0054 as calculated by unpaired t-test. B) Mean viral titer of L2 and macrophage passage 10 viral populations in macrophages, 24 hours post-infection at MOI=0.01. Statistical significance was determined by unpaired t-test ($p<0.0001$). C) Replication of plaque purified p10 isolates in macrophages, 24 hours post-infection at MOI=0.01. Data shown is representative of three independent experiments. D) Mean viral titer of plaque purified p10 isolates in macrophages, 24 hours post-infection at MOI=0.01. Statistical significance was determined by unpaired t-test ($p<0.0001$). E-G) Replication of plaque purified p10 isolates in L2 cells, with all three experiments shown due to heterogeneity in results. Statistical testing by unpaired t-test was conducted on mean viral titer values. p=.8307 (E), p=0.0007 (F), p=0.0044 (G)

bioRxiv preprint doi: https://doi.org/10.1101/2023.10.12.561935; this version posted October 14, 2023. The copyright holder for this preprint (which was not certified by peer review) is the author/funder, who has granted bioRxiv a license to display the preprint in perpetuity. It is made available under aCC-BY 4.0 International license.

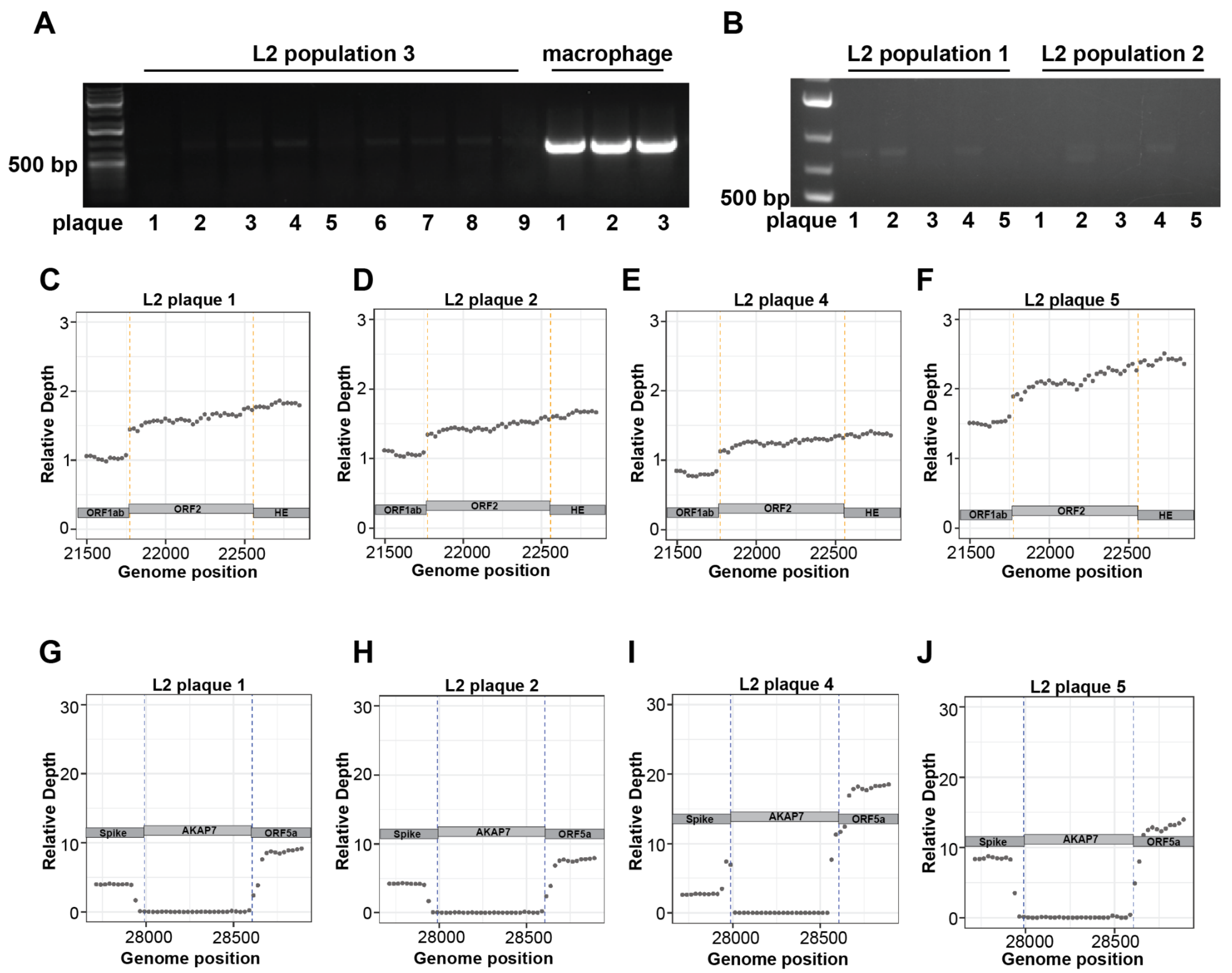

Figure S3. Direct cDNA sequencing shows loss of AKAP7 and retention of ORF2 during serial passage. A-B) PCR analysis of AKAP7 in plaque purified passage ten MHV^{AKAP7} isolates. C-F) Relative coverage depth plots of ORF in purified plaque isolates from passage 10 L2 fibroblast purified plaque isolates. G-J) Relative coverage depth plots of AKAP7 in purified plaque isolates from passage 10 L2 fibroblast purified plaque isolates.

bioRxiv preprint doi: https://doi.org/10.1101/2023.10.12.561935; this version posted October 14, 2023. The copyright holder for this preprint (which was not certified by peer review) is the author/funder, who has granted bioRxiv a license to display the preprint in perpetuity. It is made available under aCC-BY 4.0 International license.

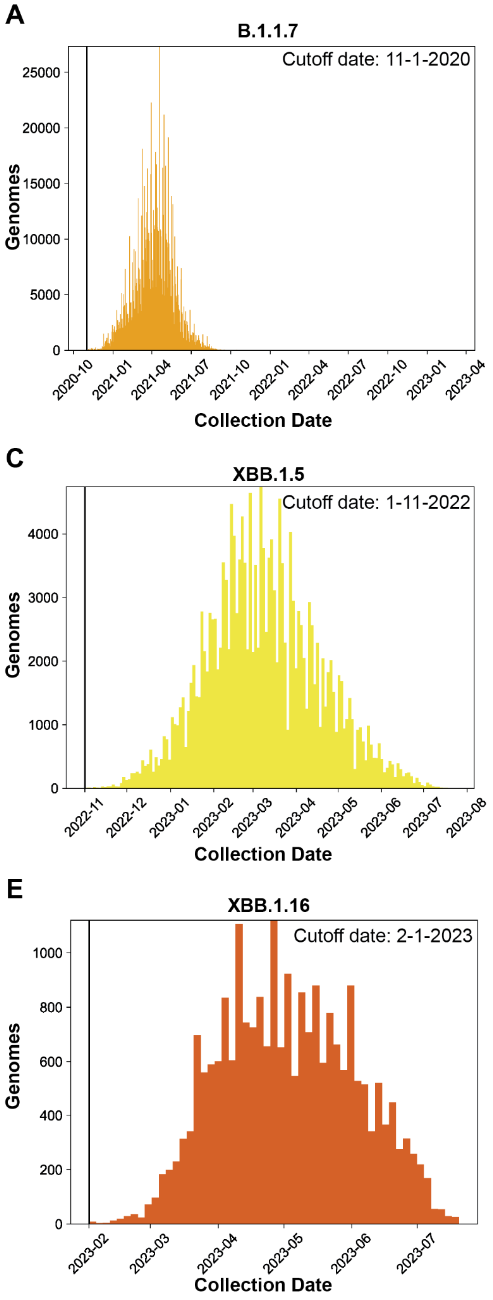

Figure S4. Histogram of ORF8 premature-stop codon SARS-CoV-2 lineage genomes plotted by sampling date. A-E) Histograms of SARS-CoV-2 lineages we sampled that have premature stop codons in ORF8. The x-axis on each plot is the collection date and the y-axis indicates the number of genomes collected on each date. The cutoff date was determined by identifying the date when sequences reported from each lineage began to increase.

bioRxiv preprint doi: https://doi.org/10.1101/2023.10.12.561935; this version posted October 14, 2023. The copyright holder for this preprint (which was not certified by peer review) is the author/funder, who has granted bioRxiv a license to display the preprint in perpetuity. It is made available under aCC-BY 4.0 International license.

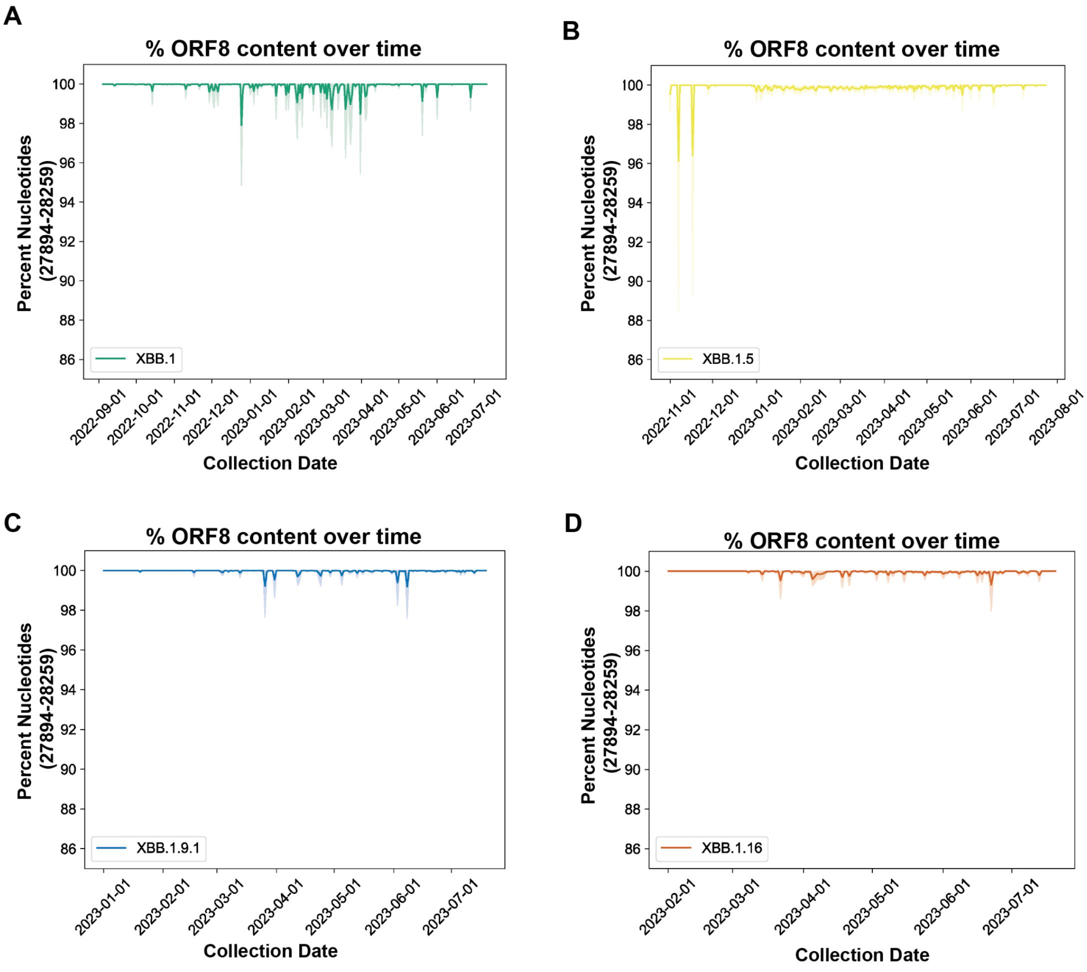

Figure S5. ORF8 gene nucleotide content over time in SARS-CoV-2 omicron sublineages. A-D) ORF8 percent nucleotide content over time in SARS-CoV-2 lineages with premature ORF8 stop codons.
